# Identification of OmpA, a *Coxiella burnetii* Protein Involved in Host Cell Invasion, by Multi-Phenotypic High-Content Screening

**DOI:** 10.1371/journal.ppat.1004013

**Published:** 2014-03-20

**Authors:** Eric Martinez, Franck Cantet, Laura Fava, Isobel Norville, Matteo Bonazzi

**Affiliations:** 1 CNRS, UMR5236, CPBS, Montpellier, France; 2 Université Montpellier 1, CPBS, Montpellier, France; 3 Université Montpellier 2, CPBS, Montpellier, France; 4 Defence Science and Technology Laboratory, Porton Down, United Kingdom; Duke University, United States of America

## Abstract

*Coxiella burnetii* is the agent of the emerging zoonosis Q fever. This pathogen invades phagocytic and non-phagocytic cells and uses a Dot/Icm secretion system to co-opt the endocytic pathway for the biogenesis of an acidic parasitophorous vacuole where *Coxiella* replicates in large numbers. The study of the cell biology of *Coxiella* infections has been severely hampered by the obligate intracellular nature of this microbe, and *Coxiella* factors involved in host/pathogen interactions remain to date largely uncharacterized. Here we focus on the large-scale identification of *Coxiella* virulence determinants using transposon mutagenesis coupled to high-content multi-phenotypic screening. We have isolated over 3000 *Coxiella* mutants, 1082 of which have been sequenced, annotated and screened. We have identified bacterial factors that regulate key steps of *Coxiella* infections: 1) internalization within host cells, 2) vacuole biogenesis/intracellular replication, and 3) protection of infected cells from apoptosis. Among these, we have investigated the role of Dot/Icm core proteins, determined the role of candidate *Coxiella* Dot/Icm substrates previously identified *in silico* and identified additional factors that play a relevant role in *Coxiella* pathogenesis. Importantly, we have identified CBU_1260 (OmpA) as the first *Coxiella* invasin. Mutations in *ompA* strongly decreased *Coxiella* internalization and replication within host cells; OmpA-coated beads adhered to and were internalized by non-phagocytic cells and the ectopic expression of OmpA in *E. coli* triggered its internalization within cells. Importantly, *Coxiella* internalization was efficiently inhibited by pretreating host cells with purified OmpA or by incubating *Coxiella* with a specific anti-OmpA antibody prior to host cell infection, suggesting the presence of a cognate receptor at the surface of host cells. In summary, we have developed multi-phenotypic assays for the study of host/pathogen interactions. By applying our methods to *Coxiella burnetii*, we have identified the first *Coxiella* protein involved in host cell invasion.

## Author Summary

Infectious diseases are among the major causes of mortality worldwide. Pathogens‚ invasion, colonization and persistence within their hosts depend on a tightly orchestrated cascade of events that are commonly referred to as host/pathogen interactions. These interactions are extremely diversified and every pathogen is characterized by its unique way of co-opting and manipulating host functions to its advantage. Understanding host/pathogen interactions is the key to face the threats imposed by infectious diseases and find alternative strategies to fight the emergence of multi-drug resistant pathogens. In this study, we have setup and validated a protocol for the rapid and unbiased identification of bacterial factors that regulate host/pathogen interactions. We have applied this method to the study of *Coxiella burnetii*, the etiological agent of the emerging zoonosis Q fever. We have isolated, sequenced and screened over 1000 bacterial mutations and identified genes important for *Coxiella* invasion and replication within host cells. Ultimately, we have characterized the first *Coxiella* invasin, which mediates bacterial internalization within non-phagocytic cells. Most importantly, our finding may lead to the development of a synthetic vaccine against Q fever.

## Introduction

*Coxiella burnetii* is an obligate intracellular Gram-negative bacterium responsible of the worldwide neglected zoonosis Q fever [1], [2]. Acute forms of the disease are characterized by a febrile illness associated with severe headache, pneumonia and hepatitis. In a small percentage (2–5%) of cases, acute Q fever develops into a chronic infection that may lead to endocarditis and chronic fatigue syndrome [1], [3]. *Coxiella* resists environmental stress by generating small cell variants (SCVs) that facilitate its airborne dissemination; during infections, this pathogen converts into a metabolically active large cell variant (LCV) with a unique resistance to the degradative machinery of host cells [4], [5]. These factors contribute to the extreme infectivity of this microbe, making of *Coxiella* a serious health concern, especially in rural areas where outbreaks are likely to occur and are accompanied by heavy economic burdens [6], [7]. Moreover, the development of *Coxiella* as a potential bioweapon during and since World War II, has ascribed this pathogen among class B biothreats [7]. *Coxiella* has two antigenic phases: phase I organisms, isolated from natural sources of infection, are extremely virulent. Phase II bacteria originate from spontaneous mutations after several *in vitro* passages of phase I organisms and present a truncated lipopolysaccharide (LPS) [8]. These non-reversible mutations result in a strong attenuation of virulence *in vivo* [9], [10]. Phase II *Coxiella* organisms are internalized more efficiently than phase I organisms by both professional macrophages and non-phagocytic cells [9], [11], however, once internalized, both antigenic phases replicate within host cells with similar kinetics. A phase II clone (Nine Mile phase II clone 4 or NMIIC4), which has been authorized for biosafety level 2 (BSL-2) manipulation, represents therefore an optimal model to study *Coxiella* infections [2], [5]. In natural infections, *Coxiella* has a tropism for alveolar macrophages [1], [2], however, infection of epithelial and endothelial cells has also been reported [12], [13]. Indeed, *in vitro*, *Coxiella* invades and replicates in a wide variety of phagocytic and non-phagocytic cells [5]. *Coxiella* internalization within host cells is a passive, endocytic process, which involves the remodeling of the host cell actin cytoskeleton [14], [15] and α_V_β_3_ integrins have been reported as *Coxiella* receptors in THP-1 cells [11]. However the *Coxiella* factors that mediate interactions with host cell surfaces, as well as the bacterial host receptor on epithelial cells remain unknown. During the first 48 hours following internalization, bacteria reside into tight-fitting vacuoles, positive for early endosomal and autophagosomal markers [16]. As *Coxiella*-containing vacuoles mature along the endocytic pathway, the drop in vacuolar pH triggers the translocation of bacterial proteins by a Dot/Icm type 4b secretion system (T4SS) [17]. Effector translocation is essential for the biogenesis of a large parasitophorous vacuole (PV) that occupies the majority of the host cytoplasm [18], [19]. Such large membranous structures are highly dynamic and fusogenic and the host endocytic SNARE Vamp7 is required for optimal PV development [20]. Importantly, mature *Coxiella* PVs are positive for lysosomal markers and contain active degradative enzymes [5], [16]. *Coxiella* infections are not lytic and bacteria-filled PVs persist within infected cells, which are protected from apoptosis by a Dot/Icm-dependent mechanism [19], [21]–[25]. Importantly, due to the obligate intracellular nature of this pathogen, the microbial factors involved in host/pathogen interactions remain to date largely unknown. The homology between the T4SS of *C. burnetii* and *L. pneumophila* allowed the *in silico* identification of 354 candidate *Coxiella* effectors based on the presence of a conserved Dot/Icm regulatory motif (PmrA) [26]–[28], C-terminal translocation signals (E-block) [26]–[28], and eukaryotic-like domains [29]–[31]. Dot/Icm–dependent secretion has been validated for 108 of these using either *Coxiella* or *Legionella* as a surrogate host [18], [26]–[31]. Recent advances in *Coxiella* axenic culture techniques [32] rendered this pathogen genetically tractable [33], allowing for the first time to couple bioinformatics analysis to morpho-functional assays and investigate the role of candidate *Coxiella* virulence determinants in intracellular replication [18], [19], [27]. To date, 20 *Coxiella* genes encoding Dot/Icm substrates have been mutated to investigate their role in *Coxiella* replication within the host [27]. Here we have set up new, integrative approaches that combine transposon mutagenesis with genomics, bioinformatics and fluorescence-based functional assays aiming at the large-scale identification of intracellular bacteria virulence factors. Our approach is designed for the simultaneous investigation of multiple key steps of *Coxiella* infections and is based on the identification and characterization of transposon-induced phenotypes. We have generated and isolated 3000 *Coxiella* transposon mutants, 1082 of which have been sequenced and screened in the present study. Our analysis revealed important insights into the functionality of the *Coxiella* Dot/Icm apparatus and revealed a variety of bacterial factors involved in 1) internalization within host cells, 2) PV biogenesis and intracellular replication, and 3) protection of the infected cell from apoptosis. By focusing our analysis on the early events of *Coxiella* infections we identified the first *Coxiella* invasin that plays an essential role in bacterial internalization by non-phagocytic cells.

## Results

### Generation of a bank of Phase II *Coxiella* mutants

**Figure 1:**
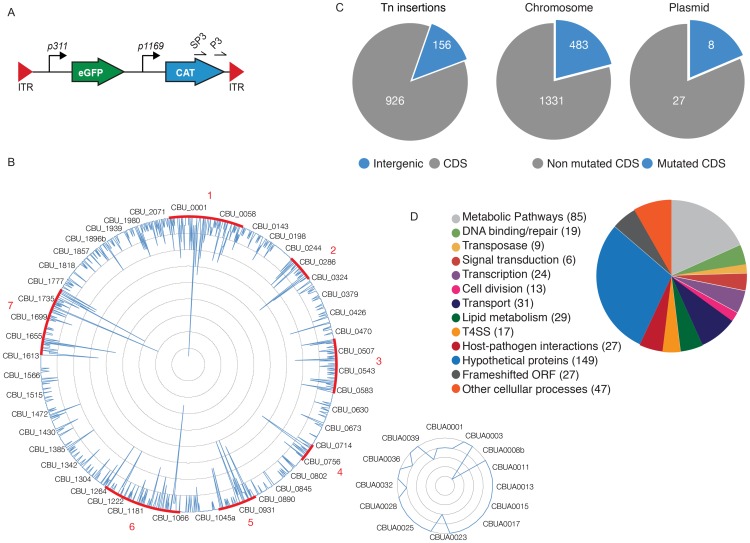
Generation of a bank of GFP-tagged *Coxiella* transposon mutants. (**A**). The transposable element used to generate the bank of *Coxiella* mutants contained the *eGFP* gene under the regulation of the strong *Coxiella* promoter *p311* and the chloramphenicol acetyltransferase gene (CAT) under the regulation of the mild *Coxiella* promoter *p1169*, all flanked by Inverted Terminal Repeats (ITR). Arrows labeled SP3 and P3 indicate the site of annealing of the corresponding primers. (**B**). Sequenced transposon insertions were annotated on the *Coxiella* RSA493 chromosome (large circle) and QpH1 cryptic plasmid (small circle). Peaks indicate the site of insertion of each transposon and the height of the peak corresponds to the frequency of mutants isolated presenting a transposon insertion in a given site (1 inner circle corresponds to 2 insertions). Sites of preferential transposon insertions are labeled in red. (**C**). Pie charts indicating the frequency of transposon insertions in *Coxiella* coding sequences (CDS) or intergenic regions of the genome (left chart) and the total number of mutated CDS as compared to the total number of CDS in the chromosome (middle chart) and cryptic plasmid (right chart). (**D**). The mutated CDS were clustered according to their predicted functions as reported by the PathoSystems Resource Integration Center (PATRIC).

To identify the *Coxiella* factors involved in host-pathogen interactions, we have undertaken the generation of a library of GFP-tagged bacterial mutants by transposon mutagenesis. We have modified the *Himar1*-based transposon system initially developed by Heinzen and colleagues [33], [34], by inserting the enhanced green fluorescent protein (*egfp*) gene under the regulation of the *Coxiella* promoter P311, upstream of the chloramphenicol resistance cassette, thus generating pITR-CAT-ColE1-P311-GFP. To obtain stable mutants, Nine Mile Phase II clone 4 (NMIIC4) *Coxiella* (hereafter referred to as *wt Coxiella*) were electroporated using a two-plasmid system, where the transposase is encoded by a suicide plasmid that is lost during bacterial replication [34]. The eGFP-tagged *Coxiella* mutants thus generated were isolated on ACCM-2 agar plates in the presence of chloramphenicol and further amplified for 7 days in liquid ACCM-2 supplemented with chloramphenicol. The final concentration of each bacterial culture was calculated using the Quant-iT PicoGreen dsDNA assay. Transposon insertion sites were identified by single-primer colony PCR followed by DNA sequencing. Using the primer SP3 we amplified DNA fragments including a 278 bp region upstream of the 3′ Inverted Terminal Repeat (ITR) of the inserted transposon (Fig. 1A). The amplified fragments were then sequenced using the transposon-specific primer P3, which recognizes a sequence in the 3′ region of the Chloramphenicol Acetyltransferase (CAT) gene (Fig. 1A). The obtained sequences were then aligned on the *Coxiella burnetii* RSA493 annotated genome using automated sequence analysis software. The genome of *Coxiella burnetii* RSA493 contains 1849 coding sequences (CDS), 1814 in the bacterial chromosome and 35 in the cryptic plasmid QpH1 [35]. To date we have isolated 3000 transposon mutants, 1082 of which have been sequenced, annotated and analyzed for this study (Fig. 1B). Transposon insertions were homogeneously distributed throughout the *Coxiella* chromosome and plasmid, with seven “hot spots” of preferential transposon insertion (identified and annotated from 1 to 7, Fig. 1B) and a large, 52 CDS region, upstream of hot spot n. 2, which remained non-mutated. Of note, region n.7 corresponds to the locus that hosts T4SS core genes (*dot*/*icm* genes) whereas the non-mutated region between CBU_0215 and CBU_0272 is enriched in genes encoding ribosomal proteins. Overall, 926 transposon insertions were found within *Coxiella* annotated CDS and 156 in intergenic regions of the *Coxiella* genome (excluding insertions within the first 100 bp upstream of a CDS; Fig. 1C). Frequency distribution analysis revealed that mutations occurred in 483 CDS on the *Coxiella* chromosome and 8 CDS on the QpH1 plasmid (corresponding to 26.6% and 22.8% of the total CDS present on chromosome and plasmid respectively; Fig. 1C). The mutated CDS were then clustered according to their predicted function based on the data available on the Pathosystems Resource Integration Center (PATRIC, www.patricbrc.org; Fig. 1D).

### High-content multi-phenotypic screen of *Coxiella* transposon mutants

**Figure 2:**
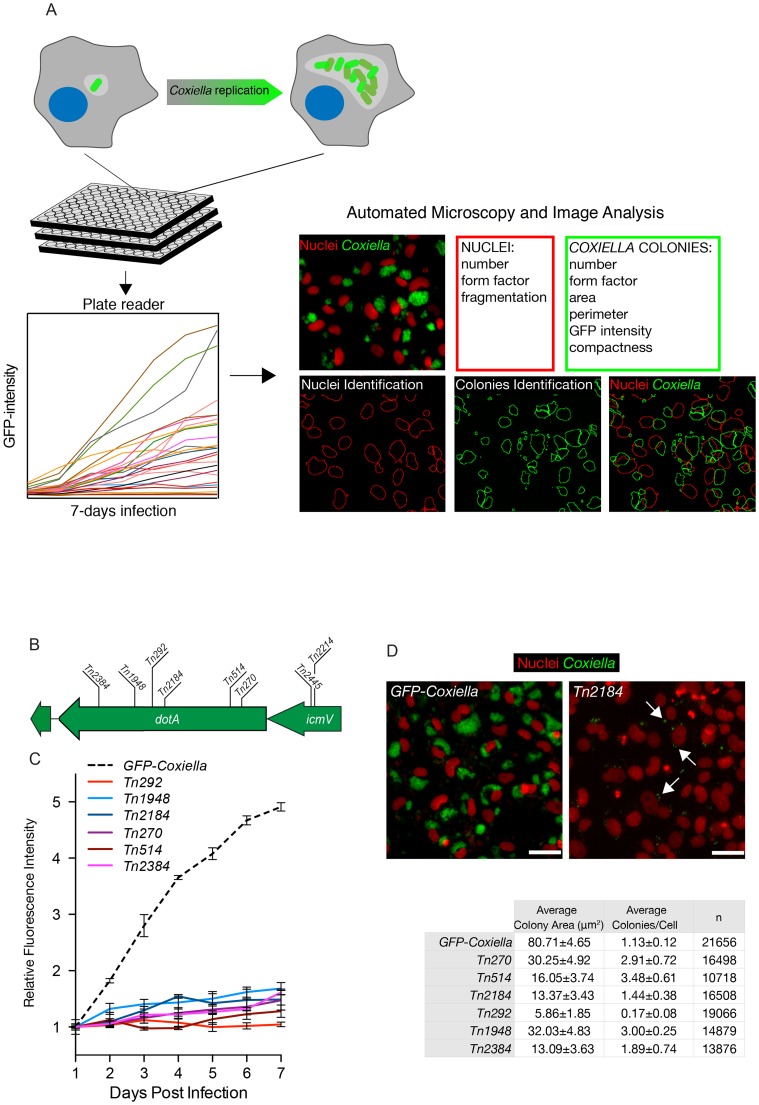
Multi-phenotypic high-content screen of *Coxiella* transposon mutants. (**A**). Schematic representation of the screening pipeline. Cells seeded in 96-well plates in triplicate were challenged with GFP-tagged *Coxiella* mutants and intracellular bacterial growth was monitored over 7 days of infection using a multimode plate reader to obtain growth curves for each mutant. Plates were then fixed, and images were acquired with an automated fluorescence microscope. The automated image analysis software CellProfiler was then used to segment the acquired images and extrapolate 3 features from infected cells (red box) and 6 features from *Coxiella* colonies (green box) for phenotype analysis. (**B**). 6 independent transposon insertions in the essential *dot/icm* core gene *dotA* were used to validate the screening approach (labeled bars indicate the sites of transposon insertion within the *dotA* and the *icmV* genes). (**C**). Intracellular growth curves of the 6 isolated *dotA* transposon mutants as compared to GFP-*Coxiella*. From day 2 post-infection, the growth curves of all *dotA* mutants were significantly different from that of GFP-*Coxiella* (P<0.001, 2way ANOVA). (**D**). Representative images of colonies from GFP-*Coxiella* and the *dotA* mutant *Tn2184* (green) juxtaposed to nuclei of infected host cells (red). Arrows point at intracellular *Coxiella*. The average area of GFP-*Coxiella* colonies was compared to that of the 6 *dotA* mutants as well as the number of colonies per cell. Data were calculated using CellProfiler; values are mean ± standard deviation of triplicate samples; the total number of analyzed cells is indicated in the right-most column of the table (n). Scale bars 20 µm.

Isolated transposon mutants were used to infect Vero cells at comparable multiplicities of infection (MOI). Non-infected Vero cells were used as negative control and cells infected with GFP-NMIIC4 *Coxiella* (GFP-*Coxiella*) [33] were used as positive controls. Variations of GFP fluorescence associated with intracellular bacterial growth over 7 days of infection were monitored using a multimode micro-plate reader to obtain intracellular growth curves for all the *Coxiella* mutants screened (Fig. 2A). Seven days after infection, plates were imaged using an automated fluorescence microscope and images were analyzed using the automated image analysis software CellProfiler (www.cellprofiler.org). 9 morphological features were extrapolated from an average of 14000 cells for each condition (Fig. 2A). Nuclei features were used to assess the overall conditions of host cells and the potential cytotoxicity of *Coxiella* mutations. *Coxiella* features were used to score the intracellular replication of bacteria. Finally, the number of *Coxiella* colonies was divided by the number of host cell nuclei to estimate the efficiency of bacterial internalization within cells.

We validated our approach by comparing the growth curves and morphology of GFP-*Coxiella* to those of 6 mutants (*Tn2384*, *Tn1948*, *Tn292*, *Tn2184*, *Tn514*, *Tn270*) carrying independent transposon insertions in the gene CBU_1648, which encodes DotA, an essential component of the *Coxiella* Dot/Icm secretion system [36] (Fig. 2B). Axenic growth of the 6 *dotA* mutants was comparable to that of *wt Coxiella* (Fig. S1A). GFP signal analysis indicated that intracellular growth of GFP-*Coxiella* followed a typical growth curve, with bacterial replication clearly detectable from day 2 post-infection and increasing until day 7 post-infection (Fig. 2C). Morphological analysis of GFP-*Coxiella*-infected Vero cells reported bacterial colonies with an average area of 80.71±4.65 microns^2^ and an average number of *Coxiella* colonies/cell of 1.13±0.12 (n = 21656; Fig. 2D). As expected, the GFP signal originated by all the DotA transposon mutants failed to increase significantly during the 7 days of infection (Fig. 2C). Accordingly, morphological analysis indicated a strong reduction in the average area of intracellular *Coxiella* colonies, which ranged from 5.86±1.85 to 32.03±4.83 microns^2^ (n = 19066 and 14879 respectively, Fig. 2D). Interestingly, the majority of *dotA* mutations corresponded to an increased number of colonies/cell, which, in the case of mutant *Tn514*, reached 3.48±0.61 (Fig. 2D). This phenotype suggests that, in the absence of a functional secretion system, the *Coxiella*-containing vacuoles fail to coalesce to form the typical, large PV. Moreover, two independent transposon insertions in the Dot/Icm gene *icmV* (*Tn2445* and *Tn2214*), which is located in the same operon as *dotA*, produced a comparable, strong replication phenotype (data not shown).

**Figure 3:**
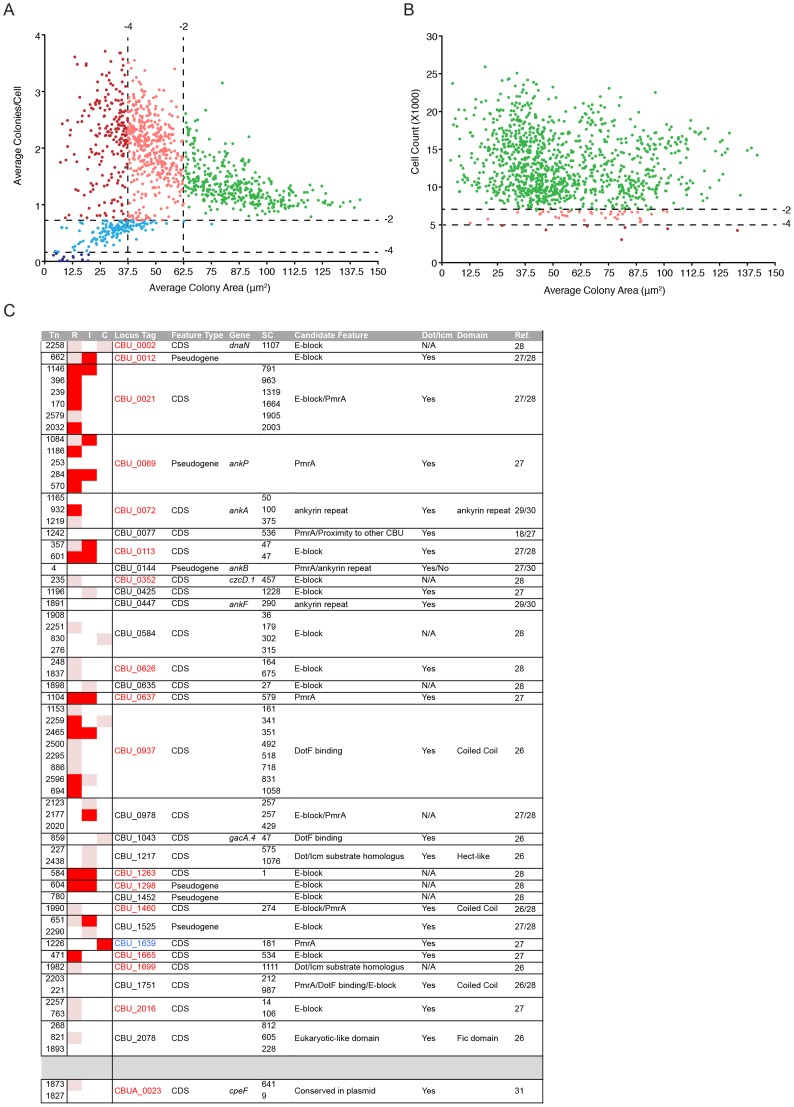
Large-scale identification of *Coxiella* factors involved in host/pathogen interactions. (**A**). For each screened mutant, the average area (in microns^2^) of *Coxiella* colonies was plotted against the relative number of colonies per cell to identify phenotypes of interest. Green dots represent phenotypes that deviate from *wt Coxiella* by a Z-score>−2 (not significant). Pink and light blue dots represent replication and internalization phenotypes respectively, with a Z-score between −2 and −4 (mild phenotypes). Red and dark blue dots represent phenotypes with a Z-score≤−4 (strong phenotypes). (**B**). For each screened mutant, the average area (in microns^2^) of *Coxiella* colonies was plotted against the total number of cells (infected and not infected) that survived 7 days of infection to estimate the cytotoxicity of transposon insertions. Green dots represent phenotypes that deviate from *wt Coxiella* by a Z-score>−2 (not significant). Pink dots represent cytotoxic phenotypes with a Z-score between −2 and −4 (mild phenotypes). Red dots represent cytotoxic phenotypes with a Z-score≤−4 (strong phenotypes). (**C**). Mutants presenting transposon insertions disrupting *Coxiella* genes previously identified as putative Dot/Icm substrates were clustered in rows according to the mutated gene (CDS) and their intracellular replication (R), internalization (I) and cytotoxic (C) phenotypes were illustrated. White squares represent non-significant phenotypes (Z-score>−2). Pink squares represent mild phenotypes (Z-score between −2 and −4). Red squares represent strong phenotypes (Z-score≤−4). Mutant number (Tn), information on the feature (Feature Type), annotated CDS name (Gene), distance of the transposon insertion site from the CDS start codon (SC), feature that allowed the identification of the gene as a candidate Dot/Icm substrate (Candidate Feature), Dot/Icm-mediated secretion (Dot/Icm), identified protein domains (Domain) and reference to previous publications (Ref.) were integrated in the table. CDS codes in red indicate significant intracellular replication phenotypes; CDS codes in blue indicate significant cytotoxic phenotypes.

The data derived from the multi-phenotypic analysis of *Coxiella* infections of host cells were mined to identify mutations that perturbed: 1) host cell invasion, 2) intracellular replication and 3) host cell survival. To screen for invasion and replication phenotypes, we plotted the average area of *Coxiella* colonies against the average number of colonies/cell (Fig. 3A). Statistical analysis was used to define regions in the resulting scatter plot corresponding to mild (−4<Z-score≤−2) and severe (Z-score≤−4) phenotypes. The 1082 analyzed *Coxiella* mutants were found in 3 well-defined clusters: one included mutants whose phenotype did not vary significantly from that of GFP-*Coxiella* (Fig. 3A green dots). A second cluster was clearly shifted towards a reduction in the size of *Coxiella* colonies and an increase in the average number of *Coxiella* colonies/cell, representative of mutations that affect *Coxiella* intracellular replication without affecting host cell invasion (Fig. 3A light and dark red dots). A third cluster was shifted towards a reduced number of colonies/cell, indicating mutations that affect host cell invasion (Fig. 3A light and dark blue dots). In parallel, the average area of *Coxiella* colonies was plotted against the total number of host cells surviving the 7 days of infection, to identify *Coxiella* genes that are potentially involved in the protection of the host cell from apoptosis (Fig. 3B). As above, statistical analysis was used to define regions corresponding to mild (−4<Z-score≤−2) and severe (Z-score≤−4) phenotypes. The vast majority of the mutants analyzed did not affect cell survival, regardless of bacterial replication within host cells (Fig. 3B green dots). 37 mutations mildly affected host cell survival (Fig. 3B light red dots), and 7 mutations were particularly detrimental to host cell survival (Fig. 3B dark red dots). Next, the phenotypic data from mutations within CDS were integrated with the annotated transposon insertions in the *Coxiella* genome. We thus clustered the screened mutations according to the mutated CDS and assigned to each mutant a color-coded map based on the intensity of their replication (R), internalization (I) or cytotoxic (C) phenotype (Table S1). Intergenic transposon insertions were retained in a separate table (data not shown).Importantly, mutant *Tn1832* carries an intergenic transposon insertion and phenocopies *wt Coxiella* and GFP-*Coxiella* (Fig. S2). This mutant has been used in our validation experiments as additional control.

### *Coxiella* factors required for intracellular replication

**Figure 4:**
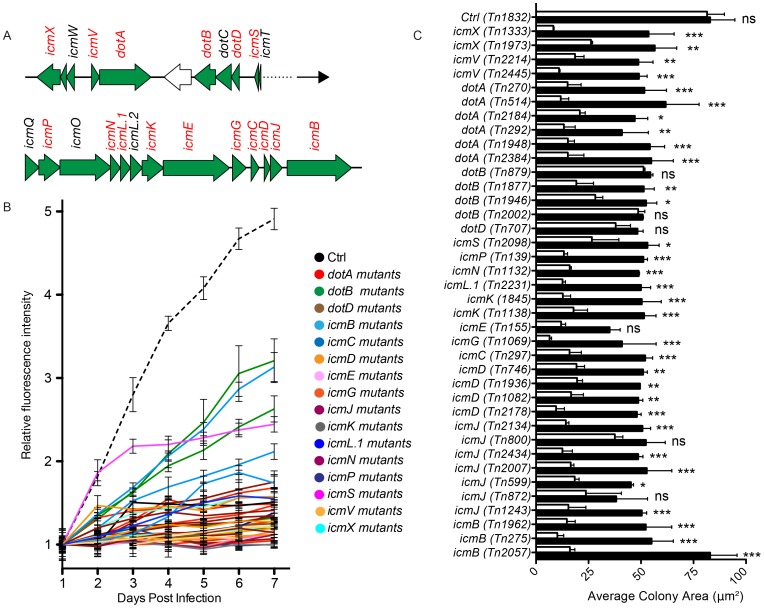
Role of Dot/Icm core proteins in *Coxiella* infections. (**A**). Schematic representation of the region of the *Coxiella* genome containing Dot/Icm genes. The genes that have been mutated in this study are represented in red. (**B**). Intracellular growth curves of *Coxiella* mutants containing transposon insertions in *dot/icm* genes calculated as variations of GFP fluorescence over 7 days of infection and compared to GFP-tagged *wt Coxiella* (Ctrl, dashed black line). Mutants in the same CDS are grouped by color. With the exception of *Tn2098* (*icmS*), the growth curves of all Dot/Icm mutants were significantly different from that of GFP-*Coxiella* (P<0.001, 2way ANOVA) from day 2 post-infection. The growth curve of *Tn2098* (*icmS*) was significantly different from that of GFP-*Coxiella* (P<0.001, 2way ANOVA) from day 3 post-infection. (**C**) Vero cells were incubated either with the Dot/Icm transposon mutants alone (white bars) or in combination with *wt Coxiella* (black bars) to complement the replication phenotypes observed. 7 days post-infection, the average area (in microns^2^) was calculated using CellProfiler from an average of 6000 cells per condition. Values are mean ± standard deviation of triplicate samples (co-infection phenotypes were compared to their respective single infection condition. ns = non-significant; * = P<0.05; ** = P<0.01; *** = P<0.001, 2way ANOVA).

Intracellular replication of *Coxiella* relies on the functionality of a Dot/Icm T4SS, which is highly homologous to that of *L. pneumophila* [2], [37]–[39]. The *Coxiella* genome contains 23 homologues to the 25 known *dot/icm* genes of the *Legionella* T4SS. Accordingly, *Legionella* has been used as a model organism to test the secretion of candidate *Coxiella* effector proteins. Recently, it has been reported that the *Coxiella dot/icm* genes *icmD*, *dotA*, *dotB* and *icmL.1* are essential for *Coxiella* replication within host cells, proving for the first time the functionality of the *Coxiella* T4SS [18], [19], [36]. The enrichment of transposon insertions in *dot/icm* genes (Fig. 1B, region n. 7), prompted us to analyze the phenotype of 38 *Coxiella* mutants carrying single transposon insertions in 16 Dot/Icm genes (Fig. 4A; Fig. S1). First, we followed the axenic growth of the 38 Dot/Icm transposon mutants and found it to be indistinguishable from that of *wt Coxiella* and of the control transposon mutant *Tn1832* (Fig. S1A). Multi-phenotypic analysis confirmed the previously reported observations that *icmD*, *dotA* and *icmL.1* are essential for *Coxiella* replication within host cells [18], [19], [36] (Fig. 4B, C; Fig. S1B). Moreover, we could observe that 12 *dot/icm* genes (*dotA*, *dotB*, *icmV, E, D, G, J, N, C, P, K, X, L.1*) are essential for bacterial replication within the host, whereas mutations in *icmB* and *icmS* showed an intermediate phenotype, which corresponded to partial intracellular replication as assessed by morphological analysis (Fig. 4B, C; Fig. S1B). Of note, transposon insertions in *dot/icm* genes present in operons produced consistent phenotypes. Each mutation was then trans complemented by challenging host cells in combination with *wt Coxiella* as previously described [19]. The intergenic mutant *Tn1832* was used as positive control. As illustrated in Figure 4C, mutations resulting in severe replication phenotypes were efficiently complemented by the presence of *wt Coxiella* in the PV occupied by Dot/Icm mutants (black bars).

We have also isolated and screened 63 transposon mutants in 31 CDS encoding predicted *Coxiella* Dot/Icm substrates (Fig. 3C). The products of 22 of these CDS have been previously reported to be positive for Dot/Icm secretion [18], [26], [27], [29], [30], [31] (Fig. 3C). Our analysis indicated that mutations in 17 of these genes produced replication phenotypes (Fig. 3C, CDS in red), further suggesting that these genes encode *Coxiella* effectors. Interestingly, a transposon insertion in CBU_1639 resulted in a strong cytotoxic phenotype (Fig. 3C CDS in blue), suggesting that this gene plays a role in the *Coxiella*-mediated inhibition of apoptosis.

The *Coxiella* genome encodes 4 two-component systems: PhoB-PhoR (CBU_0367-CBU_0366), GacA-GacS (CBU_0712-CBU_0760), QseB-QseC (CBU_1227-CBU_1228) and an RstB-like system (CBU_2005-CBU_2006) [2], [35]. In particular, the QseB-QseC system has been reported to be homologous to the *L. pneumophila* PmrA-PmrB system, a fundamental regulator of Dot/Icm secretion and its role has been indirectly confirmed [26], [40]. Consistent with these observations and with our analysis on *Coxiella* Dot/Icm genes, 6 independent transposon insertions in CBU_1227 (*qseB*) and one insertion in CBU_1228 (*qseC*), significantly impaired *Coxiella* replication within host cells (Table S1). A single transposon insertion in CBU_2006, part of the RstB-like system also produced a significant replication and entry phenotype (Table S1), however, the role of this two-component system in *Coxiella* remains to be defined.

The annotation of the *Coxiella burnetii* NMI RSA493 genome revealed the presence of 207 pseudogenes, which are not conserved among different *Coxiella* isolates [35]. Recent whole transcriptome analysis (RNA-Seq) has validated the previous annotation confirming that these sequences do not encode complete open reading frames (Prof. Howard Shuman personal communication). Interestingly, we have isolated 85 transposon mutants in 56 CDS annotated as pseudogenes and mutations in 32 of these resulted in a strong replication phenotype (Fig. S3). Finally, we could observe that 71 out of the 151 transposon insertions in intergenic regions of the *Coxiella* genome exhibited a significant replication phenotype (not shown). This may reveal small RNA-mediated regulation of *Coxiella* virulence. Indeed, 11 transposon insertions fall within intergenic regions where putative *Coxiella* sRNAs have been identified by RNA-Seq (Prof. Howard Shuman personal communication). Further investigations are currently aiming at an in-depth characterization of these new candidate factors that may regulate intracellular replication of *Coxiella*.

### *Coxiella* factors involved in subversion of apoptosis in infected cells

As mentioned above, our multi-phenotypic screen identified 7 transposon mutants that exhibited a strong cytotoxic phenotype when incubated with Vero cells (Fig. 3B, Fig. S4). To further analyze the phenotype of these mutants, we investigated their intrinsic capacity of triggering apoptosis and their potential of protecting infected cells from staurosporine-induced apoptosis. HeLa cells were preferred to Vero cells for this assay as they displayed a more consistent response to staurosporine and all *Coxiella* strains tested displayed similar replication phenotype in these cells (data not shown). Cells were either left unchallenged or incubated with *wt Coxiella*, the control transposon mutant *Tn1832*, the DotA mutant *Tn270* and the 7 cytotoxic mutants (*Tn881*, *Tn616*, *Tn946*, *Tn926*, *Tn1226*, *Tn1232*, *Tn1233*). Three days post-inoculation, cells were either fixed in paraformaldehyde or incubated with 1 µM staurosporine for 4 h prior to fixation. The percentage of apoptotic cells was then evaluated for each condition by the TUNEL assay. Very few TUNEL-positive cells were observed among untreated cells, these were increased to 50% of the total cell population upon staurosporine treatment. As expected, incubation of cells with *wt Coxiella* did not increase the number of TUNEL-positive cells as compared to untreated cells and *wt Coxiella*-colonized cells were efficiently protected from staurosporine-induced apoptosis. Cells challenged with the control transposon mutant *Tn1832* presented the same phenotype as cells incubated with *wt Coxiella* and conversely, the DotA mutant *Tn207* failed to protect infected cells from induced apoptosis (Fig. S4). Mutant *Tn881*, which carries a transposon insertion in CBU_0485, exhibited a partial protection of infected cells from induced apoptosis whereas the remaining mutants failed to effectively protect cells from the effects of staurosporine (Fig. S4). Interestingly, incubation of HeLa cells with mutants carrying transposon insertions in CBU_1639, CBU_1366 and CBU_0307a significantly increased the number of TUNEL-positive cells also in the absence of staurosporine, indicating that these mutants may possess intrinsic cytotoxic properties (Fig. S4).

### Identification of a *Coxiella* surface protein involved in host cell invasion

**Figure 5:**
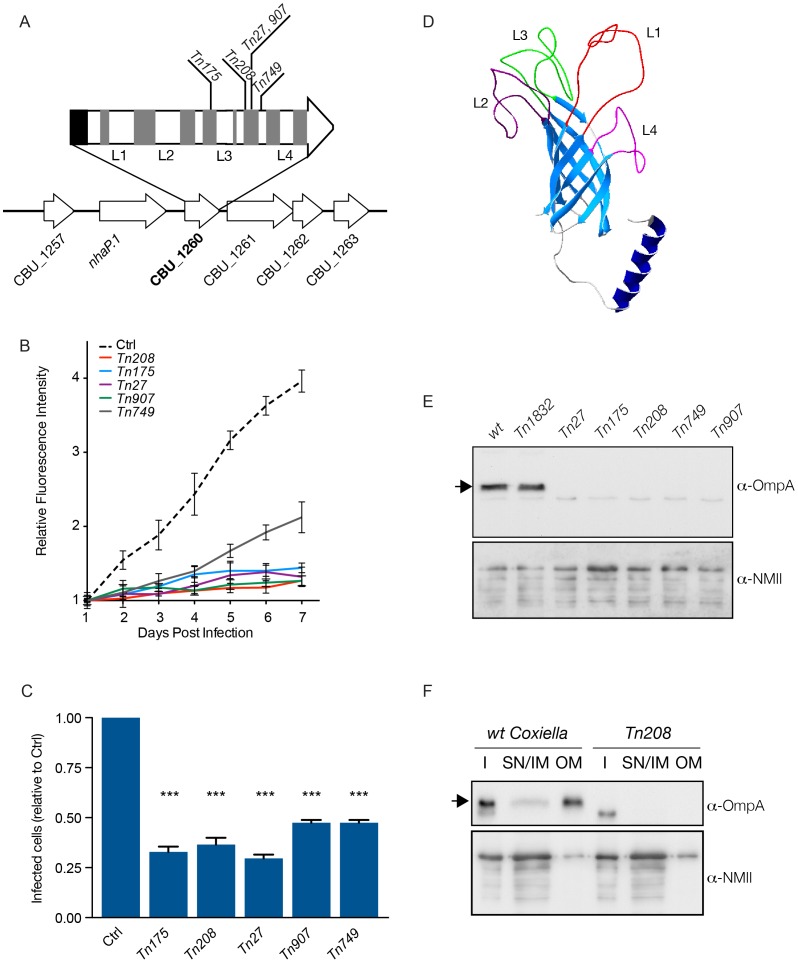
Characterization of CBU_1260 (OmpA), a *Coxiella* protein involved in host cell invasion. (**A**). Schematic representation of the genomic context of CBU_1260 and sites of transposon insertion of 5 independent isolated mutants (*Tn175*, *Tn208*, *Tn27*, *Tn907*, *Tn749*). Black area indicates the position of the predicted N-terminal α-helix and grey areas indicate the position of predicted β-sheets. Predicted extracellular loops are indicated from L1 to L4. (**B**). Intracellular growth curves of *Coxiella* mutants containing transposon insertions in CBU_1260 measured as variation of GFP fluorescence over 7 days of infection and compared to GFP-tagged *wt Coxiella* (Ctrl). The growth curves of all CBU_1260 mutants were significantly different from that of GFP-*Coxiella* (P<0.001, 2way ANOVA) from day 2 post-infection. (**C**). For each CBU_1260 mutant analyzed, the total number of *Coxiella* colonies identified by automated image analysis was divided by the total number of cell nuclei and expressed as relative to GFP-tagged *wt Coxiella* (Ctrl) to derive the efficiency of host cell invasion. Values are mean ± standard deviation of triplicate samples where an average of 12000 cells were analyzed per condition (values were compared with Ctrl condition. *** = P<0.001, 2way ANOVA). (**D**). iTASSER-derived prediction the CBU_1260 product. The central core of the protein is composed of an 8-β-sheet transmembrane barrel domain (light blue). The putative periplasmic domain is composed of a α-helix (dark blue), whereas the predicted extracellular domain is composed of four (L1 to 4) unstructured loops. (**E**). Representative Western blot of bacterial lysates from *wt Coxiella*, the control mutant *Tn1832* and the 5 mutants presenting transposon insertions in CBU_1260. (**F**). Representative Western blot of *wt Coxiella* and mutant *Tn208* subjected to membrane fractionation to assess the localization of OmpA (I = insoluble fraction; SN/IM = supernatant and inner membrane fraction, OM = outer membrane fraction). Blots were revealed with an antibody raised against the first extracellular loop (L1) of OmpA (top panels) and with a polyclonal antibody against *Coxiella* NMII (bottom panels) used as loading control. The arrows indicate the expected size of OmpA (23 kDa).

As for all obligate intracellular pathogens, *Coxiella* invasion of host cells is a priming step of the infection. However, since the bacterial factors that mediate *Coxiella* invasion of host cells remain unknown, we sought to identify bacterial factors whose mutations affect *Coxiella* internalization. High-content screening identified 48 mutations in 37 *Coxiella* CDS that resulted in a significant reduction in the number of infected cells after 7 days of infection (Fig. S5). Of these, 18 CDS involved in bacterial metabolism and transcription were excluded. Among the remaining 19 candidate CDS (Fig. S5, CDS boxed in red), we have identified 5 independent transposon insertions (*Tn175*, *Tn208*, *Tn27*, *Tn907 and Tn749*) in CBU_1260, all sharing a consistent, strong internalization phenotype (Fig. S5). CBU_1260 is a 747 bp CDS on the positive strand of the *Coxiella* chromosome encoding a hypothetical protein of a predicted size of 23 kDa. Importantly, the gene is not part of an operon, indicating that the phenotype observed for the 5 mutants analyzed in this study was indeed due to the inactivation of CBU_1260 alone (Fig. 5A). Axenic growth of the 5 transposon mutants was comparable to that of *wt Coxiella* and of the control transposon mutant *Tn1832* (Fig. S6A). As expected, intracellular growth curve analysis of the 5 transposon mutants in CBU_1260 indicated that GFP fluorescence failed to increase during the 7 days of infection (Fig. 5B). Indeed, all 5 mutations in CBU_1260 reduced the number of cells presenting *Coxiella* colonies at 7 days of infection by 60–70% compared to cells challenged with GFP-*Coxiella* (Fig. 5C). We next used the online analysis software i-TASSER (http://zhanglab.ccmb.med.umich.edu/I-TASSER/) and Phyre2 (http://www.sbg.bio.ic.ac.uk/phyre2/) to predict the structure of the hypothetical protein encoded by CBU_1260. Bioinformatics analysis predicted 8 transmembrane beta sheets forming a beta barrel domain, an N-terminal alpha helix and 4 unstructured loops (L1 to L4), exposed at the cell surface (Fig. 5A, D). This prediction was confirmed by analyzing the sequence of the protein using TMpred (http://www.ch.embnet.org/software/TMPRED_form) and the BetAware software [41]. Sequence analysis indicated that transposon insertions occurred in the distal part of the CDS, within the 4^th^ and 6^th^ beta sheets, with mutants *Tn27* and *Tn907* presenting insertions at the same site (Fig. 5A). The predicted transmembrane domain of CBU_1260 is typical of Outer Membrane Protein A (OmpA) family of proteins, which are found in several bacteria and mediate adhesion and/or internalization within host cells [42]–[50]. We therefore named the product of CBU_1260 OmpA. *Coxiella* encodes 3 hypothetical proteins that contain predicted OmpA-like domains: CBU_0307, CBU_0936 and CBU_1260 (*ompA*). Sequence alignment of these three hypothetical proteins showed high degree of homology at the level of the transmembrane domains and the N-terminal alpha helix (Fig. S7). However, little homology was observed at the level of the 4 unstructured loops of OmpA (Fig. S7). Accordingly, a transposon insertion in CBU_0307 produced a cytotoxic phenotype whereas 4 transposon insertions in CBU_0936 produced a replication phenotype (Table S1). An OmpA-specific antibody was then raised against the predicted extracellular domain of the protein. To this aim, a 15 amino acid peptide in predicted loop 1 (KKSGTSKVNFTGVTL) was used for its immunogenic potential as compared to peptides in the other loops. When tested by Western blot on lysates from *wt Coxiella* and the control mutant *Tn1832*, the anti-OmpA antibody revealed a major band at the expected size of 23 kDa (Fig. 5E, arrow) and a faint, background band of lower molecular weight (Fig. 5E). When incubated on lysates from the 5 transposon mutants in CBU_1260, the anti-OmpA antibody only recognized the background band of lower molecular size. We next performed membrane fractionation assays on *wt Coxiella* and the OmpA mutant *Tn208* to validate the outer membrane localization of OmpA. The protein was highly enriched in the outer membrane fraction of *wt Coxiella* and, as expected, absent in lysates from the OmpA mutant *Tn208* (Fig. 5F). Taken together, our data indicate that CBU_1260 encodes an outer membrane protein with a predicted OmpA-like structure that plays a relevant role in host cell invasion.

### OmpA mediates *Coxiella* internalization and intracellular replication within host cells

**Figure 6:**
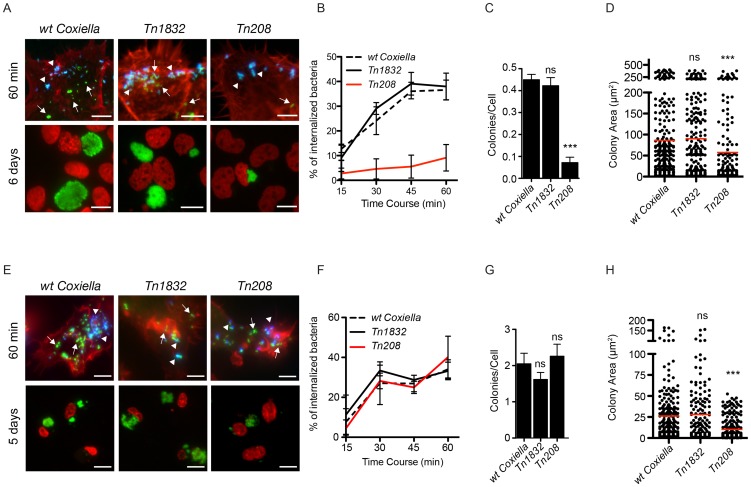
*Coxiella* OmpA is involved in host cell invasion and intracellular replication. A431 (**A**) and THP-1 (**E**) cells were incubated with *wt Coxiella*, the control transposon mutant *Tn1832* or the OmpA mutant *Tn208*. 60 minutes after infection (top panels) cells were fixed and labeled with an anti-*Coxiella* antibody coupled to Alexa Fluor 555 (blue) and with Atto-647N phalloidin (red) prior to cell permeabilization. Internalized bacteria were detected by GFP fluorescence (green) in the case of *Tn208* and *Tn1832* whereas for *wt Coxiella* infections, cells were permeabilized and bacteria were stained with the anti-*Coxiella* antibody as above, coupled to Alexa Fluor 488 (green). Alternatively, cells were fixed 6 (**A**, bottom panels) or 5 (**E**, bottom panels) days after infection, DNA (red) was labeled with Hoechst 33258 and *wt Coxiella* (green) with the specific antibody as above. The automated image analysis software CellProfiler was used to calculate the percentage of internalized bacteria (**B** and **F**), the number of colonies/cell (**C** and **G**) and the area (in microns^2^) of intracellular *Coxiella* colonies identified for each condition (**D** and **H**). Values are means ± standard deviations of triplicate experiments where an average of 8000 bacteria (B and F) or 400 vacuoles (C, D, G, H) were analyzed for each condition (values were compared to *wt Coxiella* infections. ns = non-significant; *** = P<0.001 2way ANOVA for B and F and t test for C, D, G, H). The difference between the percentage of internalized *wt Coxiella* (or the control mutant *Tn1832*) and the *Tn208* mutant was statistically significant in **B** (P<0.001, 2way ANOVA) and non-significant in **F**. Arrows indicate internalized bacteria (green); arrowheads indicate extracellular bacteria (green and blue). Scale bars 10 µm.

To further investigate the role of OmpA in *Coxiella* invasion of host cells, we validated the transposon insertion in the OmpA mutant *Tn208* by PCR and used a GFP-probe to confirm by Southern blot that *Tn208* contained a single transposon insertion (Fig. S6B, C). Next, non-phagocytic epithelial cells (A431), THP-1 (PMA-differentiated), J774 and RAW 264.7 macrophages were challenged either with *wt Coxiella*, the OmpA mutant *Tn208* or the control transposon mutant *Tn1832*. Differential labeling of extracellular and intracellular bacteria was used to assess the efficiency of *Coxiella* internalization within host cells at 15, 30, 45 and 60 minutes post-infection (Fig. 6A, E, top panels). Longer time points (5 and 6 days post-infection) were analyzed to investigate the intracellular development of internalized OmpA mutants (Fig. 6A, E, bottom panels). Automated image analysis was then used to analyze approximately 8000 bacteria per condition and quantify the efficiency of bacterial internalization as well as the area occupied by intracellular *Coxiella* colonies. In A431 non-phagocytic cells, the internalization of *Tn208* was strongly reduced as compared to that of *wt Coxiella* or *Tn1832*, which shared similar kinetics (Fig. 6B). Interestingly, when the same internalization experiment was performed using macrophages, the three bacterial strains tested (*wt Coxiella*, *Tn208* and *Tn1832*) were internalized with comparable efficiency (Fig. 6F; Fig. S8A, D), with a concomitant local rearrangement of the actin cytoskeleton (Fig. 6E, top panels). Of note, despite the strong inhibition of bacterial internalization in A431 cells, OmpA mutants retained the capacity to adhere to host cells (Fig. 6A). This suggests that if OmpA plays a role in bacterial adhesion, this may be masked by the presence of alternative factors involved in *Coxiella* adhesion to host cells. At longer time points of infection we observed a decrease in the number of *Tn208* colonies per cells in A431 cells as compared to *wt Coxiella* and *Tn1832* colonies, which confirmed our previous observations in Vero cells (Fig. 6C). On the contrary, the number of *Coxiella*-colonized cells was not affected when macrophages were challenged either with *wt Coxiella*, the control mutant *Tn1832*, or the OmpA mutant *Tn208* (Fig. 6G, Fig. S8B, E). Remarkably however, the average area of OmpA mutant *Coxiella* colonies was significantly reduced as compared to *wt Coxiella* and *Tn1832*, regardless of the cell line used for the experiment (Fig. 6D, H; Fig. S8C, F). Interestingly, this is in agreement with previously reported roles of OmpA proteins in intracellular survival of bacterial pathogens [43], [45], [46], [51]–[53].

### OmpA is necessary and sufficient to mediate internalization within non-phagocytic cells

**Figure 7:**
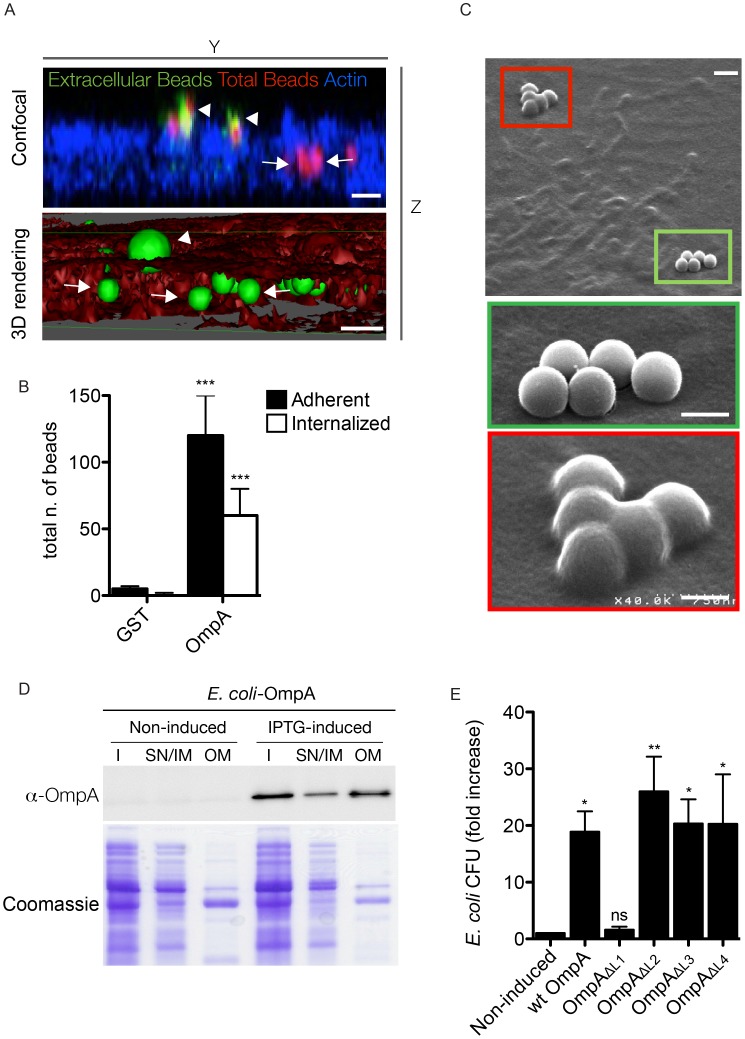
OmpA is necessary and sufficient to trigger the internalization of beads and extracellular bacteria within non-phagocytic cells. (**A**). A431 cells were incubated 60 minutes with Cy3 latex beads coated with His-tagged OmpA_32-248_ (red). To visualize extracellular beads, cells were labeled without permeabilization with an anti-histidine antibody coupled to Alexa Fluor 488 (green) and the cell cytoskeleton was labeled with Atto-647N phalloidin. Successive images were acquired along the Z-axis of the sample using a confocal microscope and the YZ view was reconstructed using ImageJ (Confocal). Alternatively, surface volume rendering (3D) was obtained using Osirix. Beads were pseudocolored in green and the cell volume in red. Arrowheads and arrows point at extracellular and intracellular beads, respectively. Scale bars 1 µm. (**B**). A431 cells were incubated 60 minutes with Cy3 latex beads coated with either His-tagged OmpA_32-248_ or GST. Extracellular beads were visualized by labeling non-permeabilized cells with an anti-histidine or an anti-GST antibody, respectively. The automated image analysis software CellProfiler was used to identify and count intracellular and extracellular (adherent) beads. Values are means ± standard deviations of triplicate experiments (values were compared to GST control conditions. *** = P<0.001, 2way ANOVA) (**C**). Representative image of A431 cells incubated 60 minutes with Cy3 latex beads coated with His-tagged OmpA_32-248_ and processed for scanning electron microscopy. Green box and inset indicate adherent beads; red box and inset indicate internalized beads. Scale bars 1 µm (top panel) and 0.5 µm (insets). (**D**). *E. coli* BL21-DE3 star pLysS transformed with pET27b-OmpA (*E. coli*-OmpA) were left untreated (Non-induced) or induced with IPTG (IPTG-induced) to synthesize periplasm-targeted OmpA. Insoluble bacterial fractions (I), supernatant and inner membrane fractions (SN/IM) and outer membrane fractions (OM) were probed with the anti-OmpA antibody by Western blot (upper panel). Samples stained with Coomassie blue were used as loading control (lower panel). (**E**). *E. coli* transformed with pET27b-OmpA (*wt* OmpA) or one of the 4 variants carrying loop substitutions (OmpA_ΔL1_, OmpA_ΔL2_, OmpA_ΔL3_, OmpA_ΔL4_) were left untreated or induced with IPTG and incubated with A431 cells. The gentamicin survival assay was used to determine the capacity of *E. coli* expressing *wt* OmpA and its mutated variants to invade non-phagocytic cells. Values are mean ± standard deviations of triplicate experiments (values were compared to non induced condition. ns = non-significant; * = P<0.05; ** = P<0.01, 2way ANOVA).

To further dissect the role of OmpA in *Coxiella* interaction with host cell surfaces, we 1) purified the recombinant protein to coat inert latex beads and 2) ectopically expressed OmpA in *E. coli*, to assess the capacity of OmpA to confer adhesiveness and invasiveness to inert particles and extracellular bacteria, respectively. Histidine-tagged, recombinant OmpA was produced by *E. coli* BL21-DE3 star pLysS transformed with the pET28a vector containing *ompA_32-248_*. The first 31 amino acids of OmpA corresponding to the intracellular N-terminal alpha helix were excluded to increase the solubility of the protein. Red fluorescent latex beads were then coated with 100 µg/ml His-OmpA_32-248_ or GST as control and used to challenge A431 cells for 1 hour at 37°C. Unbound beads were removed by rinsing cells in PBS and cells were fixed in paraformaldehyde. Cells were then probed with an anti-histidine antibody without permeabilization to differentially label adherent and internalized beads and with fluorescent phalloidin to define the cell perimeter and volume. Alternatively, after fixation samples were further processed for scanning electron microscopy. Confocal microscopy analysis of cross sections of cells incubated with His-OmpA_32-248_-coated beads revealed a fraction of beads adhering to the cell surface, hence positive for the anti-histidine staining, and another fraction within the cell volume (as defined by the actin labeling) and negative to the anti-histidine staining (Fig. 7A). Three-dimensional reconstruction of confocal sections coupled to surface rendering confirmed the presence of adhering and internalized beads (Fig. 7A). GST-coated beads failed to adhere to and invade A431 cells significantly (Fig. 7B). Scanning electron microscopy analysis corroborated these observations: several His-OmpA_32-248_-coated beads were adhering to the surface of A431 cells (Fig. 7C, green inset) whereas others were clearly covered by the cell plasma membrane (Fig. 7C, red inset). Very few GST-coated beads were observed at the surface of cells and none seemed to be internalized (data not shown), confirming our observations by fluorescence microscopy. We next assessed the capacity of OmpA to trigger the internalization of non-invasive bacteria by non-phagocytic cells using the gentamicin protection assay. To this aim *E. coli* BL21-DE3 star pLysS were transformed with pET27b-OmpA, which allowed the IPTG-regulated expression and periplasmic targeting of full-length OmpA. The expression and outer membrane localization of OmpA were verified by Western blot using the OmpA-specific antibody on transformed *E. coli* cultures induced overnight with IPTG and processed to separate the bacterial outer membranes from the inner membranes and cytoplasmic components (Fig. 7D). Non-induced, transformed bacteria were used as control. We could observe that IPTG-induced bacteria efficiently produced OmpA, which was enriched in the outer membrane fraction of *E. coli* lysates (Fig. 7D). Importantly, induction of OmpA expression conferred *E. coli* a 20-fold increase in invasiveness as compared to the non-induced bacteria (Fig. 7E). Next, we used the *E. coli* ectopic expression approach to dissect the role of the 4 predicted extracellular loops of OmpA. By replacing each loop with a myc tag, we generated 4 OmpA mutants (OmpA_ΔL1_, OmpA_ΔL2_, OmpA_ΔL3_, OmpA_ΔL4_) that were used to test their capacity to confer invasiveness to *E. coli* in a gentamicin protection assay. Similar to *wt* OmpA, all mutated proteins were detected in the outer membrane fraction of induced *E. coli* (not shown). Interestingly, only the exchange of loop 1 with a myc tag significantly reduced *E. coli* internalization by non-phagocytic cells (Fig. 7E). Collectively, these data suggest that OmpA is necessary and sufficient to mediate *Coxiella* internalization within non-phagocytic cells and that loop 1 is primarily involved in interacting with a potential cognate receptor at the surface of host cells.

### Blocking OmpA interactions with the host cell inhibits *Coxiella* internalization

**Figure 8:**
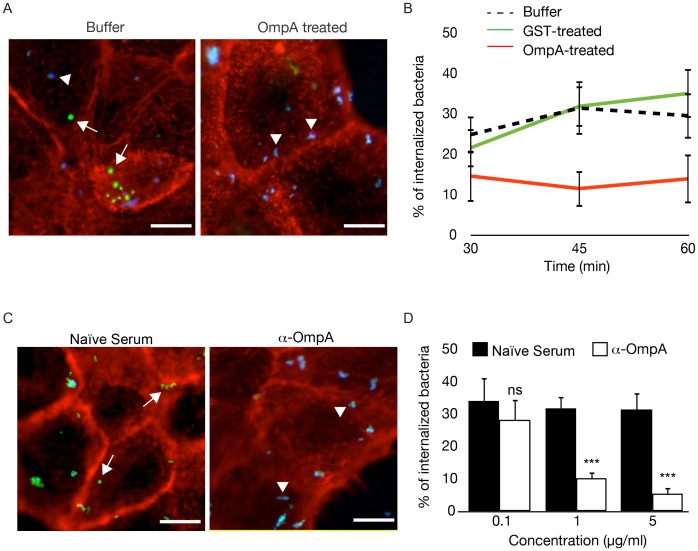
Recombinant OmpA and anti-OmpA antibody perturb *Coxiella* interactions with the host cell surface. (**A**). Representative images of A431 cells pretreated with OmpA_32-248_ buffer or with 100 µg/ml His-tagged OmpA_32-248_ and incubated with *wt Coxiella* for 60 minutes. Cells were fixed and labeled with an anti-*Coxiella* antibody coupled to Alexa Fluor 555 (blue) and with Atto-647N phalloidin (red) prior to cell permeabilization. Internalized bacteria were detected by using the same anti-*Coxiella* antibody as above coupled to Alexa Fluor 488, on permeabilized cells (green). Arrowheads and arrows point at extracellular and intracellular bacteria, respectively. (**B**). The automated image analysis software CellProfiler was used to analyze cells pretreated with OmpA_32-248_ buffer, 100 µg/ml GST or 100 µg/ml His-tagged OmpA_32-248_ and incubated with *wt Coxiella* for 30, 45 and 60 minutes. For each condition, intracellular and extracellular bacteria were automatically identified, counted and the percentage of internalized bacteria was calculated. (**C**). Representative images of A431 cells incubated for 60 minutes with *wt Coxiella* pretreated with 1 µg/ml rabbit naïve serum or anti-OmpA antibody. After fixation, cells were treated as in **A**. Arrowheads and arrows point at extracellular and intracellular bacteria, respectively. (**D**). CellProfiler was used to analyze cells incubated for 60 minutes with *wt Coxiella* pretreated with naïve serum or anti-OmpA antibody at increasing concentrations as indicated. For each condition, intracellular and extracellular bacteria were automatically identified, counted and the percentage of internalized bacteria was calculated. In all cases, values are means ± standard deviations of triplicate experiments where an average of 6000 bacteria were analyzed for each condition. In **B**, the difference between the percentage of internalized *wt Coxiella* in cells treated with buffer alone (or with GST) and cells treated with recombinant OmpA was statistically significant from 45 min of infection (P<0.001, 2way ANOVA). In **D**, values were compared to naïve serum condition. ns = non-significant; * = P<0.05; *** = P<0.001, 2way ANOVA. Scale bars 10 µm.

To determine whether OmpA function requires the interaction with host cell surface factors, we sought to block candidate ligand/receptor interactions, either by saturating potential OmpA receptors at the surface of host cells or by masking OmpA at the surface of bacteria, prior to infection. In the first case, A431 cells were incubated with 100 µg/ml His-OmpA_32-248_for 1 hour at 4°C prior to challenging with *wt Coxiella* and the efficiency of bacterial internalization was determined by differential bacterial labeling. A431 cells incubated in the same conditions with GST or with buffer alone were used as controls. Indeed, pretreating A431 cells with His-OmpA_32-248_ effectively inhibited *wt Coxiella* internalization as compared to buffer- or GST-treated cells (Fig. 8A, B). Alternatively, *wt Coxiella* were incubated with increasing concentrations (0.1, 1 and 5 µg/ml) of either anti-OmpA antibody or naïve rabbit serum prior to infection and bacterial differential labeling was used to determine *Coxiella* invasiveness in A431 cells. Confirming our previous observations, the pre-treatment of bacteria with the anti-OmpA antibody, but not naïve rabbit serum, inhibited *Coxiella* internalization in a concentration dependent manner (Fig. 8C, D). These observations suggest the presence of a receptor for OmpA at the surface of host cells, which remains to be identified, and that OmpA/receptor interactions are essential to mediate *Coxiella* internalization within host cells.

### OmpA mutation attenuates *Coxiella* virulence in the *in vivo* model system *Galleria mellonella*

**Figure 9:**
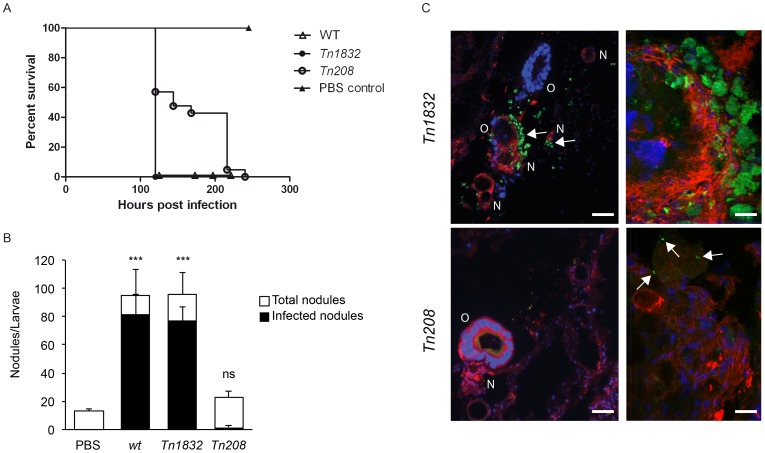
OmpA mutation perturbs *Coxiella* infections *in vivo*. (**A**). Survival chart of *Galleria mellonella* larvae injected with either *wt Coxiella* (empty triangles), the control transposon mutant *Tn1832* (full circles) or with the OmpA mutant *Tn208* (empty circles). PBS-injected larvae were used as control (full triangles). Values are means of three replicates, each with 10 injected larvae per condition. P<0.05. (**B**). The total number of nodules (white bars) and the fraction of infected nodules (black bars) from *Galleria mellonella* larvae treated as in **A** were quantified. Values are means ± standard deviations from 3 larvae analyzed per condition (values were compared to PBS condition. ns = non-significant; *** = P<0.001, 2way ANOVA). (**C**). Representative images of the larval nodules (N) and organs (O) from *Galleria mellonella* larvae treated as in **A**, fixed and processed for immuno-histochemistry. Samples were stained with Hoechst 33258 (blue), Atto-647N phalloidin (red) and an anti-GFP antibody (green). Arrows indicate the presence of *Coxiella* in nodules and organs. Scale bars 50 µm (left panels) and 20 µm (right panels).

Larvae of the wax moth *Galleria mellonella* are an emerging, efficient model for the study of host/pathogen interactions *in vivo*. Like other insects, *Galleria* larvae present essential aspects of the innate immune response to microbial infections, which are conserved in mammals. In particular, insects possess humoral and cellular defense responses, the first including antimicrobial peptides (galiomycin, gallerimycin and lysozyme in the case of *Galleria*) and the latter consisting of specialized phagocytic cells, known as hemocytes or granulocytes [54]–[57]. Importantly, in the case of several bacterial pathogens, typical phenotypes observed in mammalian infection models were efficiently reproduced using *Galleria* [58]–[61]. It has been recently demonstrated that the *Coxiella* closely-related pathogen *Legionella pneumophila* invades *Galleria* hemocytes and replicates within large membranous compartments that present the same morphology and characteristics of *Legionella*-containing vacuoles generated during infection of macrophages and amoeba [62]. Infections by *L. pneumophila* result in severe damage to insect organs, which is accompanied by an immune response, including larvae melanization and nodule formation [62]. Moreover, the role of bacterial virulence factors previously characterized in higher mammalian models is conserved during infections of *Galleria mellonella* [61], [62]. Importantly, *Galleria mellonella* larvae are also susceptible to phase II *Coxiella* infections (Norville *et al.* Unpublished data). We thus investigated the phenotype associated with the OmpA mutation carried by *Tn208* in the context of *Galleria* infections. Larvae were exposed to *Coxiella* by injecting 10^6^ bacteria (either *wt Coxiella*, *Tn1832* or *Tn208*) in the upper right proleg and larvae were incubated at 37°C up to 300 h post-infection to determine survival rates. Alternatively, larvae were incubated up to 24 and 96 h prior to fixation in paraformaldehyde and processing for immunofluorescence. In all cases, larvae injected with PBS were used as a negative control. Larvae injected with PBS alone did not show any survival defect throughout the incubation time, whereas larvae infected with *wt Coxiella* or the control mutant *Tn1832* died significantly faster compared to those infected with the OmpA mutant *Tn208* (Fig. 9A). Immunofluorescence analysis revealed that at 96 h post-inoculation, *wt Coxiella* as well as the control mutant *Tn1832* organisms triggered the formation of large, highly infected nodules of hemocytes (Fig. 9B). These were often juxtaposed to larval organs that also appeared severely infected and damaged (Fig. 9C, top panels). In contrast, when *Galleria* were challenged with the OmpA mutant *Tn208* fewer nodules of smaller size were observed throughout the larvae and only a small fraction of these presented signs of infection (Fig. 9B). When these nodules were observed at higher magnification, we could detect small *Tn208* colonies (Fig. 9C bottom panels) that were reminiscent of what we had previously observed in cultured macrophages. Our observations indicate that the OmpA-associated phenotypes observed in cultured cells can be reproduced during *in vivo* infections.

## Discussion

Infection by bacterial pathogens depends on the subversion of host functions, which is tightly orchestrated by an array of bacterial proteins referred to as virulence factors. In the last decade, cellular microbiology has stressed the importance of studying pathogens in relation to their host, however, the effective, global identification of bacterial virulence determinants and the characterization of their diverse mechanisms of action requires the development of new high-throughput and high-content screens (HTS and HCS respectively) [63]. Here, we have set up protocols for the multi-phenotypic screen of bacterial factors that are involved in host cell invasion and colonization. Our approach integrates transposon mutagenesis, genomics, bioinformatics and fluorescence-based functional assays that have been adapted for the large-scale identification of virulence factors from virtually any intracellular bacterium. The advantage of our screening technique lies in the possibility of analyzing every bacterial mutations for multiple phenotypes, such as 1) internalization within the host/cell, 2) intracellular replication and 3) cytotoxicity, simultaneously. This analysis, integrated with the map of genome mutations, allows a global overview of bacterial genes involved in host/pathogen interactions. The emerging bacterial pathogen *Coxiella burnetii* is an excellent model system to apply our strategy. To date, very little is known about the bacterial factors that regulate *Coxiella* interactions with the host; however, the recent development of axenic culture techniques now allows genetic manipulation of this microbe and in-depth analysis of its virulence factors [32], [64]. Moreover, previous *in silico* identification of putative *Coxiella* virulence factors [18], [26]–[31], provides an excellent database to cross-reference bioinformatics analysis to our functional assays. Importantly, our assay allows the identification of *Coxiella* virulence factors on a whole-genome scale, based on the phenotypes associated with the random mutagenesis of *Coxiella* CDS.

Our aim being the generation of the first bank of *C. burnetii* transposon mutants, we chose to sequence all isolated mutants independently of their phenotype during infections. This has provided a global survey of the distribution of transposon insertions and allowed us to pinpoint also those genes, suspected to encode virulence factors, which failed to produce a phenotype during *Coxiella* colonization of host cells. Mapping of transposon insertions revealed an overall homogeneous distribution of mutations, with regions of preferential transposon insertion as well as other regions that remained non-mutated. Of note, the lack of transposon insertions in the region between CBU_0678 and CBU_0698 is expected, having used the annotated genome of *Coxiella* NMI (RSA493) to map transposon insertions in *Coxiella* NMII (RSA439) in which this region is missing. Interestingly however, we failed to isolate transposon mutants from the large region between CBU_0215 and CBU_0272, enriched in essential genes encoding ribosomal proteins. This suggests that non-mutated regions may have been targeted by the transposon but gene disruption was lethal for the bacterium. Non-mutated regions may be thus exploited to map essential genes in the *Coxiella* chromosome and plasmid. When analyzing transposon mutants exhibiting a strong replication phenotype, we have occasionally observed internalization phenotypes that were not consistently reproduced in all transposon mutants isolated for a given gene. We believe that this is due to a technical limitation of our screening technique, imposed by the extremely different sizes and associated fluorescence of *Coxiella* colonies. In some cases the signal to noise ratio was very close to the threshold imposed to the image analysis software and resulted in colonies and/or bacteria that were not detected when images were segmented. Ongoing implementation of our automated analysis pipeline will allow the identification of these outlier phenotypes.

Independent studies have formally proven the essential role of the *Coxiella* Dot/Icm secretory apparatus by isolating mutants in 4 of the 23 *dot/icm Coxiella* genes (*dotA*, *dotB*, *icmD and icmL.1*) [18], [19], [36]. In all cases *dot/icm* mutants retained the capacity of invading host cells but failed to generate large PVs and replicate therein [18], [19], [36]. The enrichment of transposon insertions in the region of the *Coxiella* genome hosting *dot/icm* core genes allowed us to validate our assays exploiting existing data and, more importantly, provided a comprehensive overview of the role of the different components of the *Coxiella* T4SS. Interestingly, we have identified mutations resulting in intermediate phenotypes that allowed partial bacterial replication. Of particular interest is a mutation in *icmS*, which confers a multivacuolar phenotype to mutants. The *icmS* gene encodes a chaperone protein that mediates the secretion of a subclass of bacterial effectors [40]. The observation of a multivacuolar phenotype suggests that in *Coxiella*, IcmS may be involved in the secretion of effectors that mediate membrane fusion events required for the biogenesis of the PV. To facilitate the identification of putative IcmS substrates, a machine learning approach is currently being used to identify other transposon mutants that share the same multivacuolar phenotype.

To date, candidate *Coxiella* effectors have been identified by bioinformatics analysis based on conserved Dot/Icm regulatory motif (PmrA) [26], [27], C-terminal translocation signals (E-block) [27], [28], and eukaryotic-like domains. So far, 354 candidate effectors have been thus identified, however Dot/Icm-dependent translocation assays using either *Coxiella* or *Legionella* as a surrogate model, indicated that the majority of these might be false positives [18], [26]–[28]. In addition, the lack of efficient methods for the genetic manipulation of *Coxiella* severely hampered the functional study of these putative effectors. In a recent study, transposon mutants were obtained from 20 *Coxiella* candidate effectors with 10 exhibiting a significant replication phenotype [27]. Here we report the entry, replication and cytotoxic phenotype of 63 transposon mutants in 31 previously identified *Coxiella* candidate Dot/Icm substrates. Indeed, some of these candidates play a role during infection whereas some others fail to produce a phenotype, stressing the importance of coupling high-content screens to *in silico* analysis to identify bacterial effectors. Moreover, further studies of other *Coxiella* genes sharing none of the features of Dot/Icm substrates, can be exploited to enrich existing databases for the bioinformatics-based identification of *Coxiella* effectors, thus creating a feedback loop that would significantly improve and accelerate the study of *Coxiella* pathogenesis.

A considerable number of transposon insertions were mapped outside *Coxiella* CDS. By excluding mutations that affected the first 100 bp upstream of annotated genes (to exclude mutations that might affect promoter regions of genes), we obtained a list of 151 intergenic transposon insertions. Interestingly, 71 of these resulted in a significant replication phenotype, suggesting that these non-coding regions of the *Coxiella* genome may play a role in host/pathogen interactions. sRNAs are emerging as regulators that enable pathogens to adapt their metabolic needs during infection and timely express virulence factors [65], [66]. However, recent studies in other organisms revealed the existence of a number of putative sRNAs higher than initially expected, suggesting the presence of many non-functional sRNAs and complicating the identification of relevant sRNAs. The functional data obtained by our screening approach are being cross referenced with a list of putative *Coxiella* sRNAs identified by RNA-seq to facilitate the identification of *Coxiella* sRNAs that may coordinate host/pathogen interactions. Similarly, the interesting observation that a number of mutations in *Coxiella* CDS annotated as pseudogenes have an effect in host cell infection suggests that these genomic regions may have an important regulatory role.

Bacterial adhesion and invasion of host cells is a fundamental step of the infection by intracellular bacterial pathogens [67]. These processes can be active or passive depending on the nature of the pathogen. “Triggering” bacteria commonly use a type 3 secretion system (T3SS) to inject effectors across the host cell plasma membrane to trigger actin rearrangements and pathogen internalization by phagocytosis, whereas “zippering” bacteria use surface proteins that interact with cognate receptors at the surface of host cells [67]. This activates a ligand/receptor signaling cascade that leads to the internalization of large particles by an endocytosis-like mechanism [68]–[71]. The lack of a T3SS in *Coxiella* suggests that these organisms adhere to and invade host cells by a zippering mechanism. Indeed, it has been reported that *Coxiella* is passively internalized by host cells by a yet undefined mechanism, which is accompanied by the local rearrangement of the actin cytoskeleton [11], [14], [15]. α_V_β_3_ integrins have been shown to mediate *Coxiella* adhesion to THP-1 cells [11], however, the lack of these integrins at the surface of epithelial cells, which are effectively colonized by *Coxiella*, suggest the presence of additional/alternative receptors. Similarly, the *Coxiella* surface determinants for host cell adhesion and invasion remain to be defined. Here we have identified the product of CBU_1260 as the first *Coxiella* invasin. Predictive analysis on the primary sequence of CBU_1260 revealed the presence of 8 beta sheets forming an OmpA-like domain highly homologous to that identified and characterized in several other bacterial pathogens [49], [50]. Examples are the OmpA proteins encoded by *E. coli* K1 [44], [47], *Yersinia pestis* [45], *Francisella tularensis* [46], *Klebsiella pneumoniae* [48] and *Shigella flexneri* [72]. These outer membrane proteins are involved in bacterial adhesion and/or internalization within host cells, as well as in the NF-κB-mediated modulation of the immune response to infection, which is required for intracellular bacterial development. Importantly, OmpA-like proteins with similar functions in bacterial adhesion and internalization have been reported in other bacterial pathogens such as *Rickettsia conorii*, *Anaplasma phagocytophilum* and *Ehrlichia chaffeensis* [73]–[75], however these proteins share no structural homology with the OmpA proteins described above. In agreement with *in silico* predictions, membrane fractionation experiments performed in *Coxiella* as well as in *E. coli* ectopically expressing OmpA, showed that the protein is indeed enriched in the outer membrane fraction of bacterial lysates. Our multi-phenotypic analysis revealed that five independent transposon insertions that disrupted CBU_1260 sequence severely affected *Coxiella* internalization and replication within host cells. The internalization phenotype was specific of non-phagocytic cells, whereas OmpA mutants were still internalized by phagocytic cells. This observation indicated that, in the absence of an active phagocytic process, OmpA is able to actively trigger *Coxiella* internalization by means of ligand/receptor interactions. Importantly however, the intracellular replication of OmpA mutants was severely affected in both epithelial and macrophage cell lines. This phenotype is in line with a reported role of OmpA proteins in facilitating bacterial survival within host cells [46], [48], [51], [53]. Importantly, OmpA-like proteins share conserved transmembrane domains but are characterized by extremely variable extracellular domains, which are unique to each pathogen, and confer specific functions [50]. OmpA was predicted to have 4 unstructured loops exposed at the bacterial surface. By replacing each loop with a myc tag, we have generated 4 OmpA mutants (OmpA ΔL1, ΔL2, ΔL3 and ΔL4) and showed that loop 1 is essential to confer invasiveness to *E. coli* ectopically expressing the OmpA mutants. Accordingly, a specific antibody against loop 1 effectively blocks OmpA function. Bioinformatics analysis indicated the presence of 2 additional OmpA-like proteins in the *Coxiella* genome, CBU_0307 and CBU_0936, sharing with OmpA a good degree of homology at the level of the transmembrane OmpA-like domain but no significant homology in the 4 extracellular loops. In line with these observations, CBU_0307 and CBU_0936 failed to produce internalization phenotypes when mutated by transposon insertions. Finally, experiments aiming at blocking potential OmpA interactions with a cognate receptor, effectively blocked *Coxiella* internalization, indicating the presence of an interacting partner at the surface of host cells, which remains to be identified. Using *Galleria mellonella* larvae as a surrogate *in vivo* model system we could reproduce the OmpA mutant phenotypes observed in cultured cells. Indeed, only *wt Coxiella* and the control mutant *Tn1832* were able to induce the formation of nodules that were abundantly colonized by *Coxiella* and disrupt the organization of *Galleria* peripheral organs. The OmpA mutant *Tn208* induced a milder formation of nodules that presented few, isolated bacteria. Accordingly, larvae infected with the OmpA mutant survived *Coxiella* infections longer than those infected with the control mutants.

In summary, multi-phenotypic screening of host/pathogen interactions is an efficient method for the study of infectious diseases. Here we have applied this method to *Coxiella* infections and identified a bacterial protein that is essential for *Coxiella* internalization within non-phagocytic cells. Understanding how intracellular bacteria adhere to and invade their host is essential to 1) understand the cell biology of infection and identify the candidate targets of anti-infectious molecules and 2) to develop targeted vaccines. Of note, bacterial OmpA proteins are considered as new pathogen-associated molecular patterns (PAMPs) and are among the most immuno-dominant antigens in the outer membrane of Gram-negative bacteria [50], [76]. Our laboratory currently investigates the possibility of using OmpA to develop a synthetic vaccine against Q fever.

## Materials and Methods

### Bacterial strains, cell lines and growth conditions

Strains used in this study are listed in Fig. S9. *Escherichia coli* strains were grown in Luria-Bertani (LB) medium supplemented with ampicillin (100 µg/ml), kanamycin (50 µg/ml) or chloramphenicol (30 µg/ml) as appropriate. *Coxiella burnetii* NMII and transposon mutants were grown in ACCM-2 [77] supplemented with kanamycin (340 µg/ml) or chloramphenicol (3 µg/ml) as appropriate in a humidified atmosphere of 5% CO_2_ and 2.5% O_2_ at 37°C. Cells were routinely maintained in RPMI (Vero, THP-1, J774 and RAW 264.7) or DMEM (A431 and HeLa), containing 10% fetal calf serum (FCS) in a humidified atmosphere of 5% CO_2_ at 37°C. For experiments, THP-1 were allowed to differentiate into macrophages for 2 days in the presence of 200 nM phorbol myristate acetate (PMA, Sigma).

### Antibodies and reagents

Hoechst 33258, rabbit anti poly-His, anti mouse and anti-rabbit HRP-conjugated antibodies and Atto-647N phalloidin were purchased from Sigma. Rabbit anti *Coxiella* NMII antibodies were kindly provided by Robert Heinzen. Synthesis and production of the peptide KKSGTSKVNFTGVTL, as well as the generation of the cognate antibody in rabbit (named anti-OmpA in this study) were performed by Eurogentec, Belgium. Mouse and rabbit IgG conjugated to Alexa Fluor 488 and 555 as well as Prolong Gold antifade mounting reagent were purchased from Invitrogen. Paraformaldehyde was provided by Electron Microscopy Sciences, PA.

### Plasmids

Plasmids and primers used in this study are listed in Fig. S9. DNA sequences were amplified by PCR using Phusion polymerase (New England Biolabs) and gene-specific primers (Sigma). To create the plasmid pITR-CAT-ColE1-P311-GFP, the promoter of CBU_0311 (P311) was amplified from *Coxiella* RSA439 NMII genomic DNA using primers P311-XhoI-Fw and P311-Rv, GFP was amplified from pEGFP-N1 (Clontech) using primers EGFP-Fw and GFP-PITR-Rv, and P1169-CAT-ColE1 was amplified from plasmid pITR-CAT-ColE1 using primers GFP-PITR-Fw and XhoI-PITR-Rv. PCR fragments P311, GFP and P1169-CAT-ColE1 were fused by overlapping PCR. The resulting PCR product was digested with XhoI and ligated to obtain circular pITR-CAT-ColE1-P311-GFP. OmpA_32-248_ was amplified from *Coxiella* RSA439 NMII genomic DNA using primers OmpA_32-248_-BamHI-Fw and OmpA-EcoRI-Rv and cloned into pET28a to obtain pET28a-OmpA_32-248_. OmpA was amplified from *Coxiella* RSA439 NMII genomic DNA using primers OmpA-BamHI-shift-Fw and OmpA-EcoRI-Rv and cloned into pET27b to obtain pET27b-OmpA. Plasmids pET27b-OmpA_ΔL1_, pET27b-OmpA_ΔL2_, pET27b-OmpA_ΔL3_ and pET27b-OmpA_ΔL4_ were generated by PCR using pET27b-OmpA as template and primer pairs loop1-myc-HindIII-Fw/loop1-myc-HindIII-Rv, loop2-myc-HindIII-Fw/loop2-myc-HindIII-Rv, loop3-myc-HindIII-Fw/loop3-myc-HindIII-Rv, loop4-myc-HindIII-Fw/loop4-myc-HindIII-Rv. The PCR products were digested with HindIII and ligated to obtain the corresponding plasmids.

### Generation of a bank of *Coxiella* transposon mutants

*C. burnetii* RSA439 NMII organisms were electroporated with the pITR-CAT-ColE1-P311-GFP and pUC19::Himar1C9 plasmids [34] using the following setup: 18 kV, 400 Ω, 25 µF. Bacteria were then grown overnight in ACCM-2 supplemented with 1% FBS and the following day 3 µg/ml chloramphenicol were added to bacterial cultures. Bacteria were then amplified for 4 days and plated on solid ACCM-2 for clone isolation. Seven days post-inoculation, colonies were isolated and amplified for 6 days in liquid ACCM-2 supplemented with 3 µg/ml chloramphenicol. The concentration of each isolated mutant was quantified using the PicoGreen (Invitrogen) assay according to manufacturer's instructions. To map transposon insertions, single primer colony PCR was performed on 1 µl of *C. burnetii* transposon mutant in stationary phase in ACCM-2. The PCR mix contained 1× HF buffer (New England Biolabs), 200 µM dNTPs, 1 µM primer SP3 and 1 U of Phusion polymerase (New England Biolabs). The PCR cycle consisted in initial denaturation (98°C, 1 min), 20 high stringency cycles (98°C, 10 sec; 50°C, 30 sec; 72°C, 90 sec), 30 low stringency cycles (98°C, 10 sec; 30°C, 30 sec; 72°C, 90 sec) and 30 high stringency cycles (98°C, 10 sec; 50°C, 30 sec; 72°C, 90 sec) followed by a final extension at 72°C for 7 min. PCR products were then sequenced at Beckman Coulter Genomics (Stansted, UK) using primer P3. Insertion sites were mapped on the annotated *C. burnetii* RSA493 NMI genome using MacVector (MacVector Inc.) and recorded in a relational database (FileMaker).

### Axenic growth of *Coxiella*

10^6^ GE/ml of bacteria were inoculated in 4 ml ACCM-2 and allowed to grow for 8 days in a humidified atmosphere of 5% CO_2_ and 2.5% O_2_ at 37°C. Where needed, 3 µg/ml chloramphenicol were added to bacterial cultures. At the indicated time points bacterial concentrations were evaluated from 100 µl of cultures, using the PicoGreen (Invitrogen) assay according to manufacturer's instructions.

### Mutant library screening

Vero cells were seeded into triplicate 96-wells plates (Greiner Bio one) 2 days prior to infection. Cells were then challenged with *C. burnetii* RSA439 NMII transposon mutants at an MOI of 100. For each plate, cells in well A1 were left uninfected and cells in wells A2 and A3 were incubated with GFP-*C. burnetii* RSA439 NMII at multiplicities of infection of 100 and 200. Bacterial contact with cells was promoted by centrifugation (10 min, 400 g, RT) and cells were incubated in a humidified atmosphere of 5% CO_2_ at 37°C. Unbound bacteria were removed after 1 h of incubation and cells were further incubated in fresh culture medium for 7 days. Plates were analyzed at a 24-hours interval using a TECAN Infinite 200 Pro operated by the Magellan software (TECAN) to monitor the variations of GFP fluorescence associated with the intracellular growth of *Coxiella*. Raw data were analyzed for background subtraction, normalization and quality control among triplicates using in-house developed methods. Seven days after infection, plates were fixed in 3% paraformaldehyde in PBS at room temperature for 30 minutes, rinsed in PBS and incubated in blocking solution (0.5% BSA, 50 mM NH_4_Cl in PBS, pH 7.4). Cells were then incubated in Hoechst 33258 diluted 1∶200 in blocking solution for 30 minutes at room temperature, rinsed and incubated in PBS. Images were acquired with an Arrayscan VTI Live epifluorescence automated microscope (Cellomics) equipped with an ORCA ER CCD camera. 6 fields/well of triplicate 96-wells plates were imaged with a 20× objective in the GFP, DAPI and Bright-field channels. Images were then processed and analyzed using CellProfiler. Briefly, the GFP channel was subtracted from the corresponding DAPI channel to avoid false identification of large *Coxiella* colonies as host cell nuclei, images were thresholded using the Otsu global method and host cell nuclei as well as *Coxiella* colonies were identified and segmented. The number, form factor and fragmentation of host cell nuclei and the number, form factor, area, perimeter, GFP intensity and compactness of *Coxiella* colonies were then calculated per object and per image. An average of 14000 cells per condition (infection with a given *Coxiella* mutant) were thus analyzed. Raw data were processed for background subtraction, normalization and quality control among the 6 fields per well and plate triplicates using in-house developed methods. Data were recorded in a relational database (FileMaker) that allowed clustering of phenotypes according to the annotated transposon insertions.

### Immunofluorescence staining and microscopy

Cells were fixed in 3% paraformaldehyde in PBS at room temperature for 30 minutes, rinsed in PBS and incubated in blocking solution (0.5% BSA, 50 mM NH_4_Cl in PBS, pH 7.4). When appropriate, 0.05% saponin was added to the blocking solution for cell permeabilization. Cells were then incubated with the primary antibodies diluted in blocking solution for 1 h at room temperature, rinsed five times in PBS and further incubated for 45 min with the secondary antibodies diluted in the blocking solution. Fluorescent phalloidin was added to the secondary antibodies to label actin, where needed. After labeling, coverslips were mounted using Prolong Gold antifade mounting medium (Invitrogen) supplemented with Hoechst 33258 for DNA staining. For differential labeling, extracellular bacteria or beads were stained using specific antibodies without permeabilizing the cells. Intracellular bacteria or beads were visualized by green and red fluorescence, respectively. Alternatively, a second staining was performed after cellular permeabilization. Secondary antibody labeling using two different fluorochromes (before and after permeabilization) allowed discrimination between adherent extracellular bacteria/beads and those that have been internalized. Samples were analyzed with a Zeiss Axioimager Z1 epifluorescence microscope (Carl Zeiss) connected to a Coolsnap HQ^2^ CCD camera. Images were acquired alternatively with 63× or 40× oil immersion objectives and processed with MetaMorph (Universal Imaging Corp.). Image J and CellProfiler software were used for image analysis and quantifications. 3D reconstruction and surface rendering were performed using the Osirix software.

### Complementation assay

Transposon insertions in Dot/Icm core genes were complemented in trans as previously described [19]. Briefly, Vero cells grown on 96-wells plates were either challenged with the transposon mutants alone or in combination with *wt Coxiella* at a 1∶1 ratio for a total MOI of 100. Bacterial contact with cells was promoted by centrifugation (10 min, 400 g, RT) and cells were incubated in a humidified atmosphere of 5% CO_2_ at 37°C. Unbound bacteria were removed after 1 h of incubation and cells were further incubated in fresh culture medium for 7 days. Plates were then fixed in 3% paraformaldehyde in PBS at room temperature for 30 minutes, rinsed in PBS and incubated in blocking solution (0.5% BSA, 50 mM NH_4_Cl in PBS, pH 7.4). Cells were then labeled with the anti-NMII antibody to detect *wt Coxiella* and with Hoechst 33258 as described above for DNA labeling. 96-well plates were imaged and analyzed essentially as described above for the mutant library screening protocol with the additional acquisition of the TRITC channel to detect and segment *wt Coxiella* colonies. Object masking was applied using CellProfiler to specifically calculate the area of mutant *Coxiella* colonies growing within *wt Coxiella*-occupied PVs.

### TUNEL assay

The terminal deoxyribonucleotidyl transferase-mediated triphosphate (dUTP)-biotin nick end labeling (TUNEL) method was used for detection of DNA fragmentation of nuclei using the In Situ Cell Death Detection Kit TMR (Roche) according to the manufacturer's instructions. Briefly, HeLa cells grown on 96-well plates in triplicate were either left untreated or challenged with the indicated *Coxiella* strains at an MOI of 100 and incubated at 37°C for 3 days. Cells were then either fixed and permeabilized as described above or incubated with 1 µM staurosporine for 4 hours prior to fixation. Samples were then incubated 1 h at 37°C in the dark with TUNEL reaction mixture. Samples were then washed three times with PBS, and incubated with Hoechst 33258 for DNA staining and with an anti-NMII antibody to detect bacteria in the samples infected with *wt Coxiella*. 96-wells plates were analyzed with an Arrayscan VTI Live epifluorescence automated microscope (Cellomics) equipped with an ORCA ER CCD camera. 10 fields/well were imaged with a 20× objective in the GFP (bacteria), TRITC (TUNEL), DAPI (nuclei) and Bright-field (cells) channels. Images were then processed and analyzed using CellProfiler. Briefly, the GFP channel was subtracted from the corresponding DAPI channel to avoid false identification of large *Coxiella* colonies as host cell nuclei, images were thresholded using the Otsu global method and host cell nuclei, *Coxiella* colonies and fragmented nuclei were identified and segmented. The percentage of fragmented nuclei over the total number of nuclei was then calculated on an average of 6000 cells per condition.

### Scanning electron microscopy

Cells were washed in PBS and fixed with 2.5% glutaraldehyde in Sorensen buffer, pH 7.2 for an hour at room temperature, followed by washing in Sorensen buffer. Fixed samples were dehydrated using a graded ethanol series (30–100%), followed by 10 minutes in graded Ethanol-Hexamethyldisilazane and finally Hexamethyldisilazane alone. Subsequently, the samples were sputter coated with an approximative 10 nm thick gold film and then examined under a scanning electron microscope (Hitachi S4000, at CRIC, Montpellier France) using a lens detector with an acceleration voltage of 20 kV at calibrated magnifications.

### Immuno-histology

*Galleria mellonella* larvae were fixed overnight in paraformaldehyde 4% in PBS (pH 7.4). Larvae were then rinsed 3 times in PBS and cryo protected by successive incubations in PBS containing increasing concentrations of sucrose (10%, 20%, 30%). Samples were then frozen in isopentane at −80°C using a SnapFrost machine (Excilone). Consecutive 20 µm sections were then obtained from each sample using a Leica CM3050S cryostat. For indirect immuno-fluorescence, sections were permeabilized and blocked in PBS, 10% goat serum, 0.3% Triton X-100 for 1 h at room temperature. Samples were then incubated 48 hours at 4°C with the anti GFP antibody, then rinsed in PBS. Samples were then incubated with the appropriate secondary antibodies, Atto-647N phalloidin and Hoechst 33258 for 24 hours at 4°C. Samples were then washed in PBS and mounted on glass slides for microscopy analysis. Samples were analyzed either with an EVOS microscope (AMG) or with an ApoTome-equipped Zeiss Axioimager Z1 epifluorescence microscope (Carl Zeiss) connected to a Coolsnap HQ^2^ CCD camera. Images were acquired alternatively with a 10× objective (EVOS) or with a 63× oil immersion objective (Axioimager Z1) and processed with Image J and AxioVision (Carl Zeiss).

### Protein expression and purification

Genes cloned into pET27b or pET28a vectors were expressed in *E. coli* BL21-DE3 star pLysS (Invitrogen). Bacterial cultures were grown at 25°C to mid-exponential phase (OD_600 nm_ = 0.5) and were induced overnight with 400 µM isopropyl-β-D- thiogalactopyranoside (IPTG). For GST expression, *E. coli* XL1-blue were transformed with pGEX-4T1, grown at 37°C to mid-exponential phase (OD_600 nm_ = 0.5) and induced for 4 h with 1 mM IPTG. Bacteria were harvested by centrifugation, resuspended in lysis buffer (20 mM Tris pH 8, 300 mM NaCl, 5% glycerol, complete anti-protease (Roche)) and lysed with BugBuster (Novagen) following the manufacturer's recommendations. Lysates were then cleared by centrifugation (11 000 g, 20 min, 4°C). Proteins were purified by gravity flow using Ni^2+^ agarose His-select resin column (Sigma) for His-tagged proteins or glutathione-sepharose (Sigma) for GST. His-tagged and GST proteins were eluted with lysis buffer supplemented with 250 mM imidazole or 25 mM reduced glutathione, respectively.

### Membrane fractionation

For *Coxiella* membrane fractionation, 100 ml of *wt Coxiella* or *Tn208* mutant grown in ACCM-2 for 7 days were pelleted and resuspended in 200 µl 20 mM Tris pH 8 containing 1× Complete protease inhibitor (Roche). For *E. coli* membrane fractionation, 30 ml of IPTG-induced or non-induced *E. coli* BL21-DE3 star pLysS pET27b-OmpA, pET27b-OmpA_ΔL1_, pET27b-OmpA_ΔL2_, pET27b-OmpA_ΔL3_ or pET27b-OmpA_ΔL4_ were pelleted and resuspended in 5 ml 20 mM Tris pH 8 containing 1× Complete protease inhibitor (Roche). Bacteria were sonicated using a Branson Sonifier S-450 (6 pulses of 20 s at 40% intensity) and cleared by centrifugation at 10000 g for 5 min at 4°C. Inner membrane proteins were extracted by incubation with sarkosyl (0.5% final concentration) at RT for 15 min. Outer membrane proteins were pelleted by ultracentrifugation (TLA-100, 32000 r.p.m., 30 min, 4°C) and resuspended in 2× Laemmli sample buffer. Insoluble, soluble/sarkosyl-solubilized and outer membrane fractions were resolved by SDS-PAGE and analyzed by Coomassie staining (Sigma) or immunoblotting with anti-OmpA and anti NMII antibodies.

### Preparation of protein-coated beads

0.5 µm fluorescent red sulfate-modified polystyrene beads (Sigma) were washed three times with 25 mM MES pH 6.1 (MES buffer). The sulfate-modified beads (7.2×10^9^) were then mixed with either 100 µg/ml purified GST or His-OmpA_32-248_ and incubated at room temperature (RT) for 4 h. The GST- or His-OmpA_32-248_-coated beads were then washed three times with MES buffer and resuspended in MES buffer containing 1% BSA.

### Internalization assays

For fluorescent beads internalization assay, 7×10^7^ GST- or His-OmpA_32-248_-coated beads in DMEM were applied to 1×10^5^ A431 cells seeded onto glass coverslips in 24-well plates and contact was promoted by centrifugation (10 min, 400 g, RT). Cells were incubated in a humidified atmosphere of 5% CO_2_ at 37°C. Cells were washed three times with PBS and fixed in 4% paraformaldehyde before being processed for immunofluorescence staining. Bacteria internalization assays were performed as follow: 6×10^6^ *C. burnetii* NMII GE were applied to cells (MOI 100) and contact was promoted by centrifugation (10 min, 400 g, RT). Cells were then fixed in 4% paraformaldehyde before being processed for immunofluorescence staining. For protein blocking experiments, A431 cells were pre-incubated for 1 h at 4°C with either 100 µg/ml of GST or 100 µg/ml of His-OmpA_32-248_ prior to the internalization assay described above. For antibody inhibition experiments, 6×10^6^ *C. burnetii* RSA439 NMII GE were incubated at 4°C with increasing concentrations of either naïve rabbit serum or anti-OmpA antibodies (0.1 to 5 µg/ml) prior to the internalization assay described above. Gentamicin protection assays were performed as follow: IPTG-induced and non-induced BL21-DE3 star pLysS pET27b-OmpA, pET27b-OmpA_ΔL1_, pET27b-OmpA_ΔL2_, pET27b-OmpA_ΔL3_ or pET27b-OmpA_ΔL4_ were diluted to OD_600 nm_ = 0.5 in DMEM and applied to cells at an MOI of 10 and contact was promoted by centrifugation (10 min, 400 g, RT). Following 1 h incubation at 37°C/5% CO_2_, cells were washed 5 times with PBS and incubated for 2 h with DMEM supplemented with 100 µg/ml gentamicin. Cells were then washed 5 times with PBS, lysed with 1 ml 0.1% Triton X-100 in *dd*H2O and serial dilutions were plated onto LB agar plates supplemented with the appropriate antibiotic for colony-forming units (CFU) assessment. Internalization frequency was determined as the number of CFU surviving the gentamicin challenge out of the total bacterial input. The results are representative of at least three independent experiments.

## Supporting Information

Figure S1 **Role of Dot/Icm core proteins in** ***Coxiella*** **infections.** (**A**). Axenic (ACCM-2) growth of the 38 Dot/Icm transposon mutants isolated in this study. *wt Coxiella* (dashed black line) and the control transposon mutant *Tn1832* (black line) were used as controls (Ctrls). Mutants in the same CDS are grouped by color. (**B**). *Coxiella* mutants in *dot/icm* genes were clustered in rows, according to the mutated gene and intracellular replication (R), internalization (I) and cytotoxic (C) phenotypes were illustrated. White squares represent non-significant phenotypes (Z-score>−2). Pink squares represent mild phenotypes (Z-score between −2 and −4). Red squares represent strong phenotypes (Z-score≤−4). Tn: Mutant number; Locus Tag: CDS number; Gene: gene name; PATRIC: accession number (PATRIC annotation); Operon: operon number (DOOR annotation); Strand: sense vs. antisense CDS; SC: transposon insertion site from CDS starting codon (bp).
(TIF)

Figure S2 **Mutant** ***Tn1832*** **carries an intergenic transposon insertion that phenocopies** ***wt Coxiella*** **and GFP-** ***Coxiella*** **.** (**A**). Intergenic insertion sites of the miniTn7 transposon carrying the *egfp* gene (top) used to generate GFP-*Coxiella* and of the *Himar1*-based transposon in mutant *Tn1832* (bottom). (**B**). Intracellular growth curves of the *Tn1832* mutant as compared to GFP*-Coxiella* in Vero cells. (**C**). Representative images of Vero cells infected with GFP-*Coxiella*, *wt*-*Coxiella* and the *Tn1832* control mutant. Colonies (green) are juxtaposed to nuclei of infected host cells (red). The average area (in microns^2^) of colonies and the number of colonies per cell were compared for the three strains. Data were calculated using CellProfiler; values are means ± standard deviations of triplicate samples; the total number of analyzed cells is indicated in the right-most column of the table (n). Scale bars 20 µm.
(TIF)

Figure S3 ***Coxiella*** **pseudogenes mutated in this study.** Mutants presenting transposon insertions disrupting *Coxiella* genes annotated as pseudogenes were clustered in rows according to the mutated gene (CDS) and their intracellular replication (R), internalization (I) and cytotoxic (C) phenotypes were illustrated. White squares represent non-significant phenotypes (Z-score>−2). Pink squares represent mild phenotypes (Z-score between −2 and −4). Red squares represent strong phenotypes (Z-score≤−4). Where available, information on the annotated CDS name (Gene), feature (Feature), and domain (Domain) were integrated in the table.
(TIF)

Figure S4 **Characterization of the 7 cytotoxic mutants isolated in this study.** (**A**). Table indicating the CDS that were mutated in the 7 cytotoxic mutants isolated. (**B**). HeLa cells were either left non-infected (N.I.) or inoculated with *wt Coxiella*, the control transposon mutant *Tn1832*, the DotA transposon mutant *Tn207* (*dotA::Tn*) and the 7 cytotoxic transposon mutants. 3 days post-inoculation cells were either fixed (white bars, Ctrl) or treated with staurosporine for 4 hours (black bars, STS) prior to fixation. CellProfiler was used to calculate the percentage of fragmented host cell nuclei as detected using the TUNEL assay. Values are means ± standard deviations of duplicate experiments where an average of 6000 cells were analyzed for each condition (values corresponding to untreated or staurosporine-treated cells were compared to their respective non-infected conditions. ns = non-significant; *** = P<0.001, 2way ANOVA).
(TIF)

Figure S5 **Identification of** ***Coxiella*** **mutations with a strong internalization phenotype.** Mutants presenting transposon insertions with a strong internalization phenotype were clustered in rows according to the mutated gene (CDS) and their intracellular replication (R), internalization (I) and cytotoxic (C) phenotypes were illustrated. White squares represent non-significant phenotypes (Z-score>−2). Pink squares represent mild phenotypes (Z-score between −2 and −4). Red squares represent strong phenotypes (Z-score≤−4). Where available, information on the annotated CDS name (Gene), feature (Feature), pathway (Pathway class) and domain (Motif/Domain) were integrated in the table. CDS putatively involved in bacterial metabolism were excluded and the remaining CDS were boxed in red.
(TIF)

Figure S6 **Characterization of CBU_1260 (OmpA) transposon mutants.** (**A**). Axenic (ACCM-2) growth of the 5 OmpA transposon mutants isolated in this study. *wt Coxiella* (dashed black line) and the control transposon mutant *Tn1832* (black line) were used as controls. (**B**). *ompA* was amplified with specific primers from mutant *Tn208* and *wt Coxiella*. A sample without template was used as negative control (Negative). The shift in PCR product size corresponds to the transposon insertion in CBU_1260. (**C**). Mutant *Tn208* genomic DNA was either left undigested or digested with BsaHI prior to migration on agarose gel and Southern blot analysis using a fluorescent GFP probe. The band observed at the expected size of 3147 bp in the digested sample confirms the unique insertion of the transposon.
(TIF)

Figure S7 **Sequence alignment of** ***Coxiella*** **OmpA-like transmembrane domain-containing proteins.** The primary sequence of CBU_1260 (OmpA) was aligned to those of CBU_0307 and CBU_0936, two hypothetical proteins annotated as OmpA-like transmembrane domain-containing proteins. Light grey boxes indicate similarities, dark grey boxes indicate identity.
(TIF)

Figure S8 ***Coxiella*** **internalization by J774 and RAW macrophages and intracellular replication.** J774 (top charts) and RAW (bottom charts) macrophages were incubated with *wt Coxiella*, the control transposon mutant *Tn1832* or the OmpA mutant *Tn208* for the indicated time points. Cells were fixed and labeled with an anti-*Coxiella* antibody coupled to Alexa Fluor 555 and with Atto-647N phalloidin prior to cell permeabilization. Internalized bacteria were detected by GFP fluorescence in the case of *Tn208* and *Tn1832* whereas for *wt Coxiella* infections, cells were permeabilized and bacteria were stained with the anti-*Coxiella* antibody as above, coupled to Alexa Fluor 488. Alternatively, cells were fixed at 5 days after infection; DNA was labeled with Hoechst 33258 and *wt Coxiella* with the specific antibody as above. The automated image analysis software CellProfiler was used to calculate the percentage of internalized bacteria (**A** and **D**), the number of colonies/cell (**B** and **E**) and the area (in microns^2^) of intracellular *Coxiella* colonies (**C** and **F**) identified for each condition. Values are means ± standard deviations of triplicate experiments where an average of 8000 bacteria (A and D) or 400 vacuoles (B, C, E, F) were analyzed for each condition (values were compared to *wt Coxiella* infections. ns = non-significant; *** = P<0.001 2way ANOVA for A and D and t test for B, C, E, F).
(TIF)

Figure S9 **Tables of strains, plasmids and primers used in this study.**
(TIF)

Table S1 **Large-scale identification of** ***Coxiella*** **factors involved in host/pathogen interactions.** All mutants screened in this study were clustered in rows according to the mutated gene (CDS) and their intracellular replication (R), internalization (I) and cytotoxic (C) phenotypes were illustrated. White squares represent non-significant phenotypes (Z-score>−2). Pink squares represent mild phenotypes (Z-score between −2 and −4). Red squares represent strong phenotypes (Z-score≤−4). Tn: Mutant number; Locus Tag: CDS number; Feature type: CDS vs. pseudogene; Gene: gene name; Product: gene product; PATRIC ID: accession number (PATRIC annotation); Operon ID: operon number (DOOR annotation); Strand: sense vs. antisense CDS; Start: CDS starting codon position; Stop: CDS stop codon position; Length: CDS length (bp); SC: transposon insertion site from CDS starting codon (bp); Pathway name/class: metabolic pathways; Candidate Feature: presence of typical features of Dot/Icm substrates; Dot/Icm Translocation: previously reported data on Dot/Icm-mediated secretion; Domain: presence of eukaryotic-like domains; Reference: reference to previously reported studies on CDS.
(XLSX)
