# Supplementary figures and images for: Identification of OmpA, a Coxiella burnetii Protein Involved in Host Cell Invasion, by Multi-Phenotypic High-Content Screening
Source: PLoS Pathog. 2014 Mar 20;10(3):e1004013. doi: 10.1371/journal.ppat.1004013 (PMC3961360; doi:10.1371/journal.ppat.1004013)

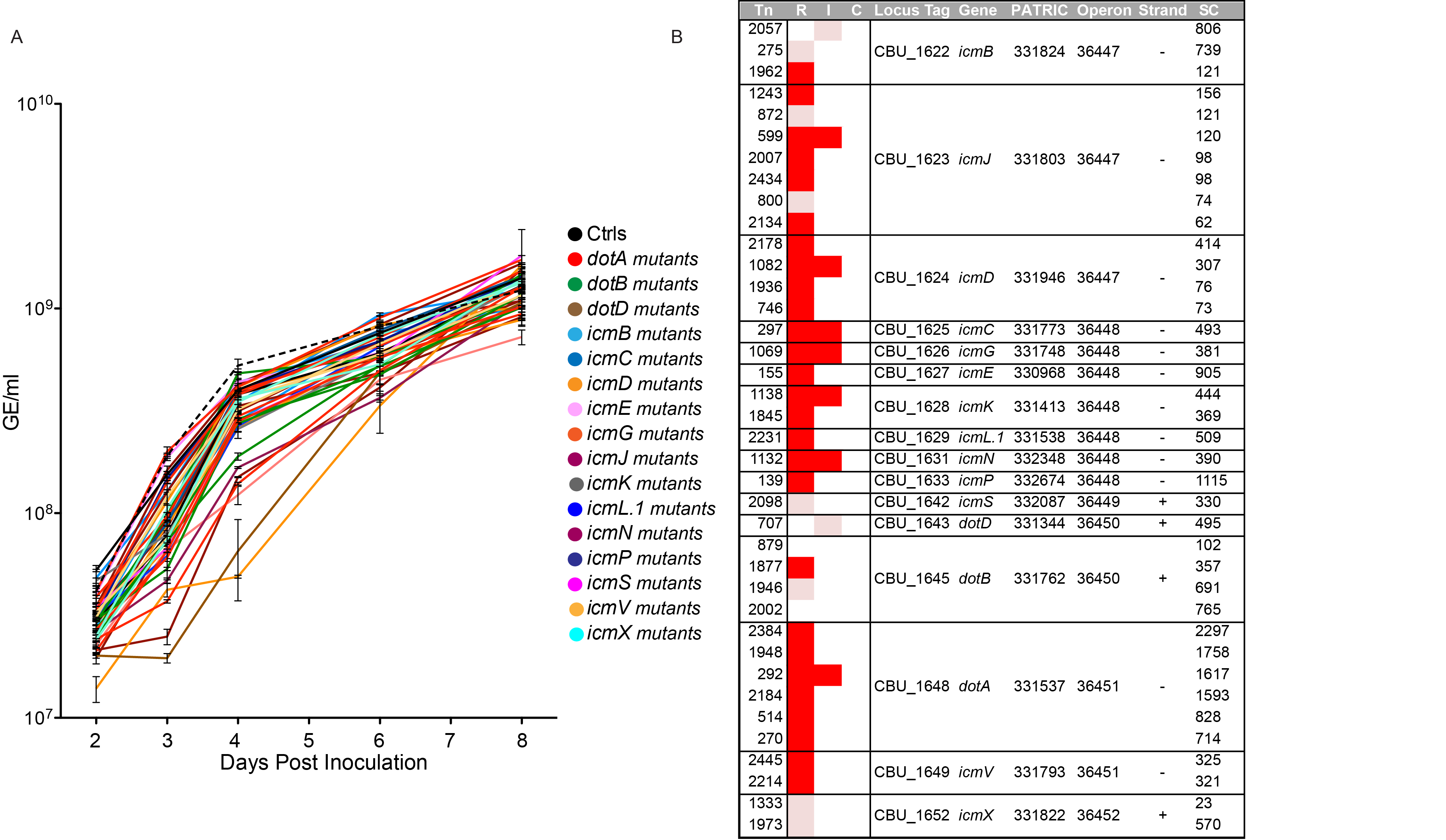

Supplement: Figure S1 — Role of Dot/Icm core proteins in Coxiella infections. (A). Axenic (ACCM-2) growth of the 38 Dot/Icm transposon mutants isolated in this study. wt Coxiella (dashed black line) and the control transposon mutant Tn1832 (black line) were used as controls (Ctrls). Mutants in the same CDS are grouped by color. (B). Coxiella mutants in dot/icm genes were clustered in rows, according to the mutated gene and intracellular replication (R), internalization (I) and cytotoxic (C) phenotypes were illustrated. White squares represent non-significant phenotypes (Z-score>−2). Pink squares represent mild phenotypes (Z-score between −2 and −4). Red squares represent strong phenotypes (Z-score≤−4). Tn: Mutant number; Locus Tag: CDS number; Gene: gene name; PATRIC: accession number (PATRIC annotation); Operon: operon number (DOOR annotation); Strand: sense vs. antisense CDS; SC: transposon insertion site from CDS starting codon (bp). (TIF) [file ppat.1004013.s001.tif]

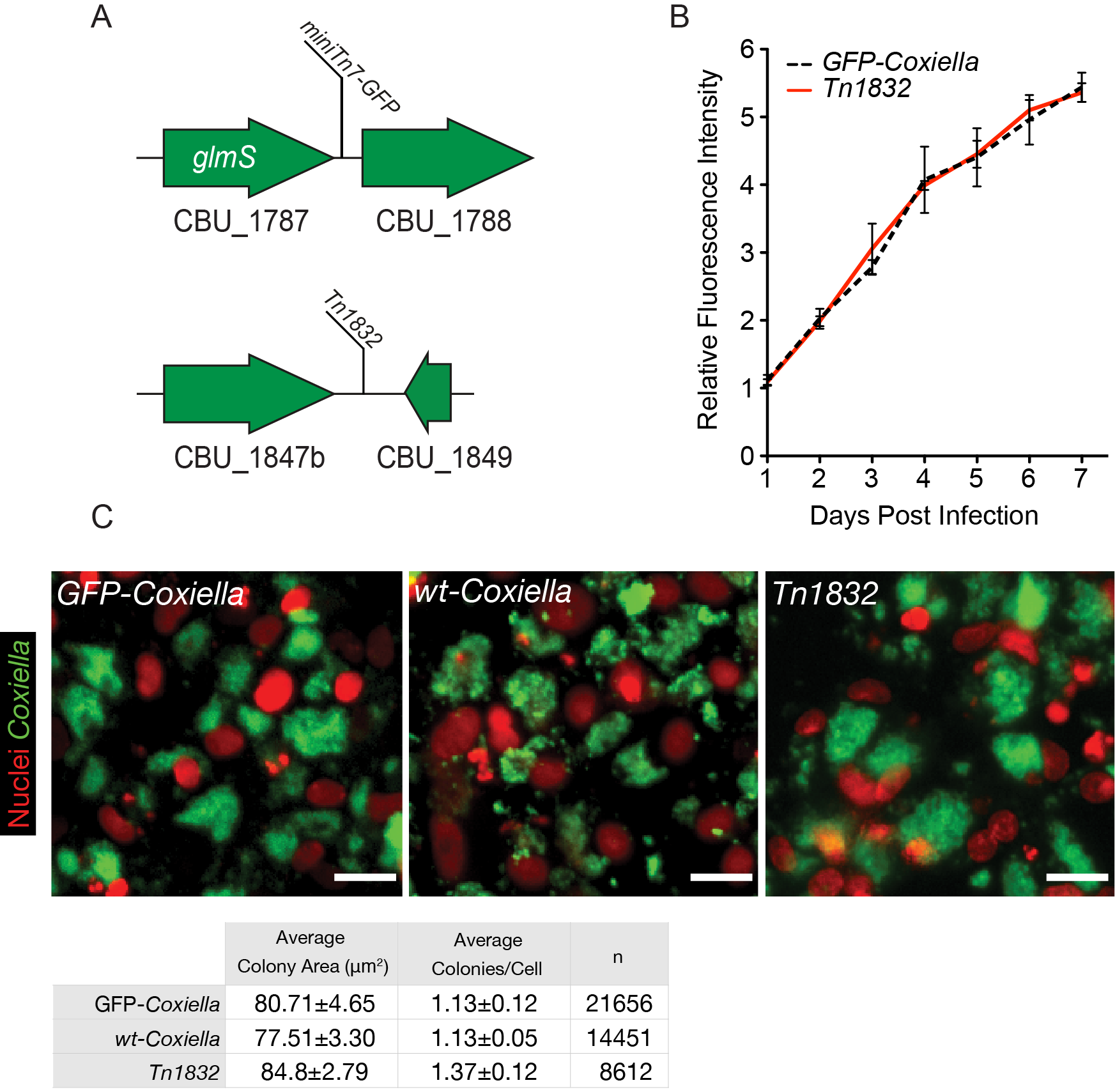

Supplement: Figure S2 — Mutant Tn1832 carries an intergenic transposon insertion that phenocopies wt Coxiella and GFP- Coxiella . (A). Intergenic insertion sites of the miniTn7 transposon carrying the egfp gene (top) used to generate GFP-Coxiella and of the Himar1-based transposon in mutant Tn1832 (bottom). (B). Intracellular growth curves of the Tn1832 mutant as compared to GFP-Coxiella in Vero cells. (C). Representative images of Vero cells infected with GFP-Coxiella, wt-Coxiella and the Tn1832 control mutant. Colonies (green) are juxtaposed to nuclei of infected host cells (red). The average area (in microns2) of colonies and the number of colonies per cell were compared for the three strains. Data were calculated using CellProfiler; values are means ± standard deviations of triplicate samples; the total number of analyzed cells is indicated in the right-most column of the table (n). Scale bars 20 µm. (TIF) [file ppat.1004013.s002.tif]

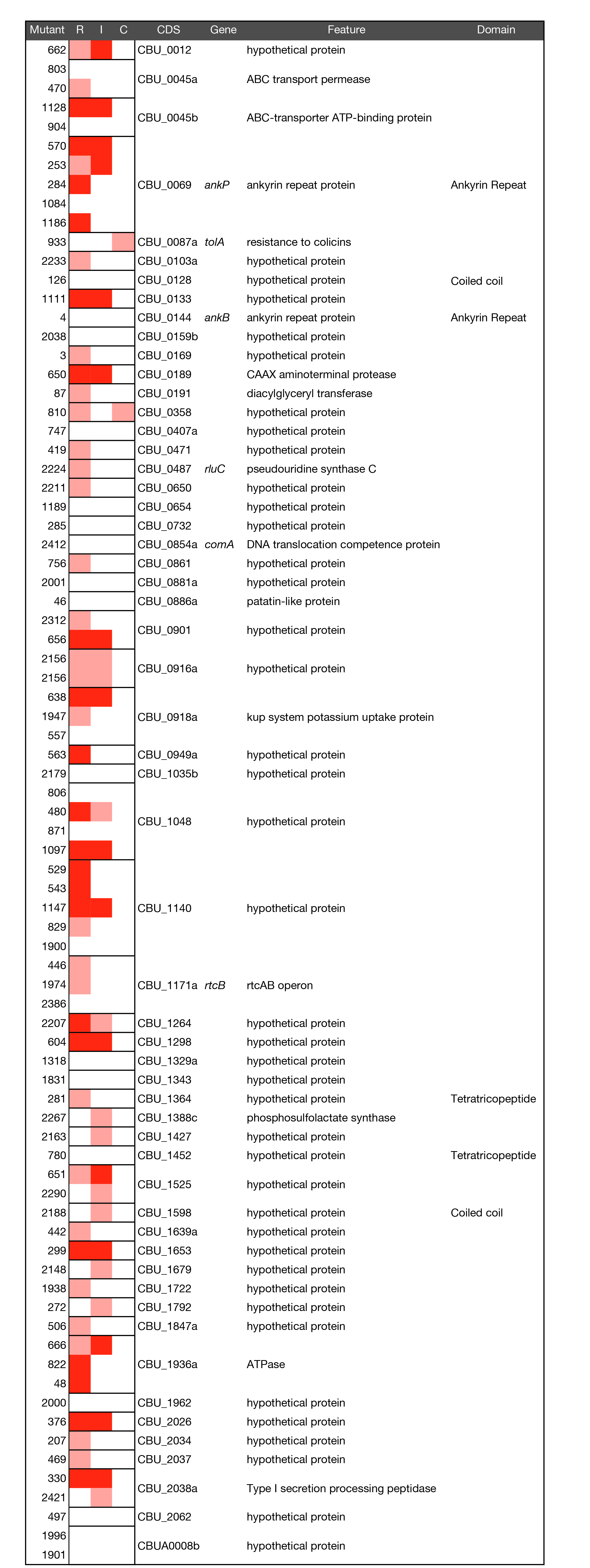

Supplement: Figure S3 — Coxiella pseudogenes mutated in this study. Mutants presenting transposon insertions disrupting Coxiella genes annotated as pseudogenes were clustered in rows according to the mutated gene (CDS) and their intracellular replication (R), internalization (I) and cytotoxic (C) phenotypes were illustrated. White squares represent non-significant phenotypes (Z-score>−2). Pink squares represent mild phenotypes (Z-score between −2 and −4). Red squares represent strong phenotypes (Z-score≤−4). Where available, information on the annotated CDS name (Gene), feature (Feature), and domain (Domain) were integrated in the table. (TIF) [file ppat.1004013.s003.tif]

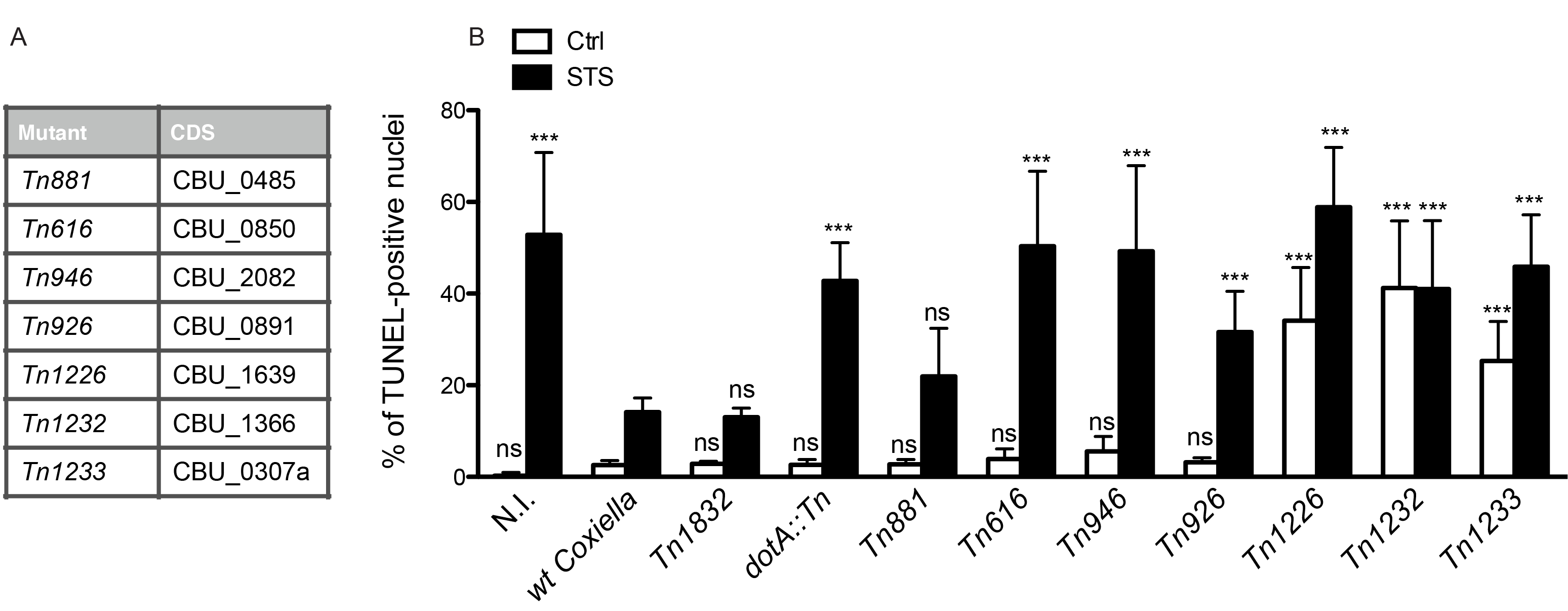

Supplement: Figure S4 — Characterization of the 7 cytotoxic mutants isolated in this study. (A). Table indicating the CDS that were mutated in the 7 cytotoxic mutants isolated. (B). HeLa cells were either left non-infected (N.I.) or inoculated with wt Coxiella, the control transposon mutant Tn1832, the DotA transposon mutant Tn207 (dotA::Tn) and the 7 cytotoxic transposon mutants. 3 days post-inoculation cells were either fixed (white bars, Ctrl) or treated with staurosporine for 4 hours (black bars, STS) prior to fixation. CellProfiler was used to calculate the percentage of fragmented host cell nuclei as detected using the TUNEL assay. Values are means ± standard deviations of duplicate experiments where an average of 6000 cells were analyzed for each condition (values corresponding to untreated or staurosporine-treated cells were compared to their respective non-infected conditions. ns = non-significant; *** = P<0.001, 2way ANOVA). (TIF) [file ppat.1004013.s004.tif]

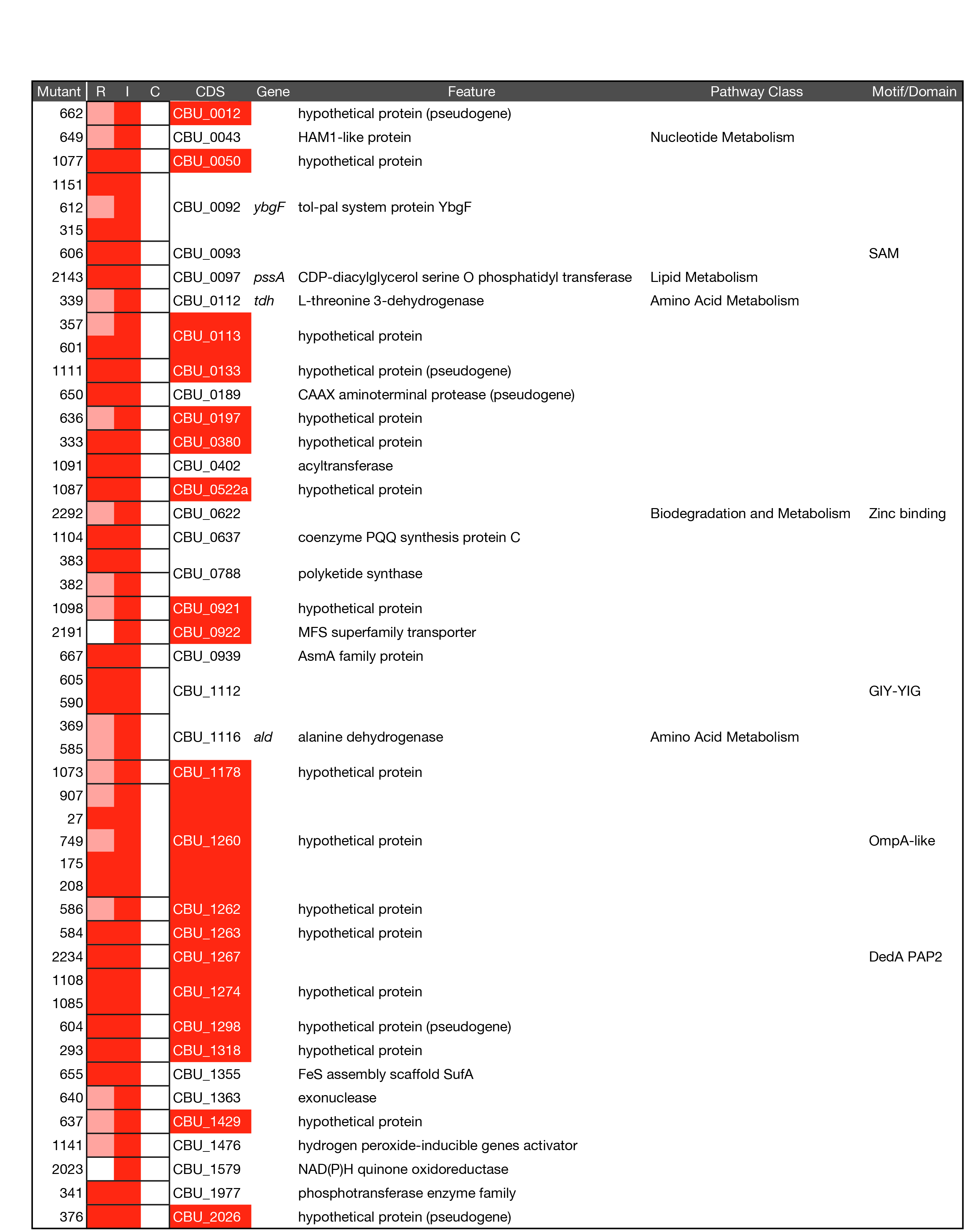

Supplement: Figure S5 — Identification of Coxiella mutations with a strong internalization phenotype. Mutants presenting transposon insertions with a strong internalization phenotype were clustered in rows according to the mutated gene (CDS) and their intracellular replication (R), internalization (I) and cytotoxic (C) phenotypes were illustrated. White squares represent non-significant phenotypes (Z-score>−2). Pink squares represent mild phenotypes (Z-score between −2 and −4). Red squares represent strong phenotypes (Z-score≤−4). Where available, information on the annotated CDS name (Gene), feature (Feature), pathway (Pathway class) and domain (Motif/Domain) were integrated in the table. CDS putatively involved in bacterial metabolism were excluded and the remaining CDS were boxed in red. (TIF) [file ppat.1004013.s005.tif]

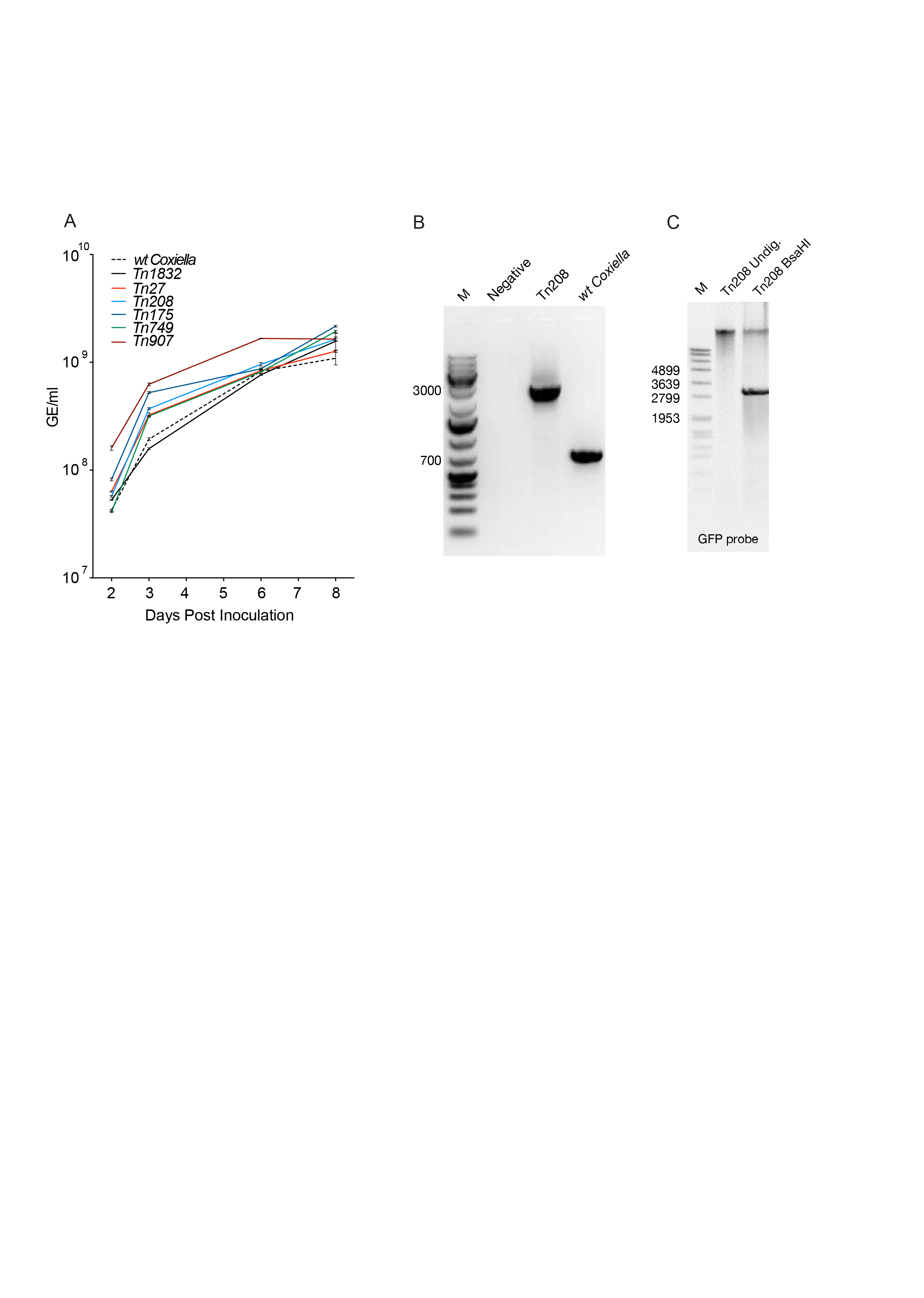

Supplement: Figure S6 — Characterization of CBU_1260 (OmpA) transposon mutants. (A). Axenic (ACCM-2) growth of the 5 OmpA transposon mutants isolated in this study. wt Coxiella (dashed black line) and the control transposon mutant Tn1832 (black line) were used as controls. (B). ompA was amplified with specific primers from mutant Tn208 and wt Coxiella. A sample without template was used as negative control (Negative). The shift in PCR product size corresponds to the transposon insertion in CBU_1260. (C). Mutant Tn208 genomic DNA was either left undigested or digested with BsaHI prior to migration on agarose gel and Southern blot analysis using a fluorescent GFP probe. The band observed at the expected size of 3147 bp in the digested sample confirms the unique insertion of the transposon. (TIF) [file ppat.1004013.s006.tif]

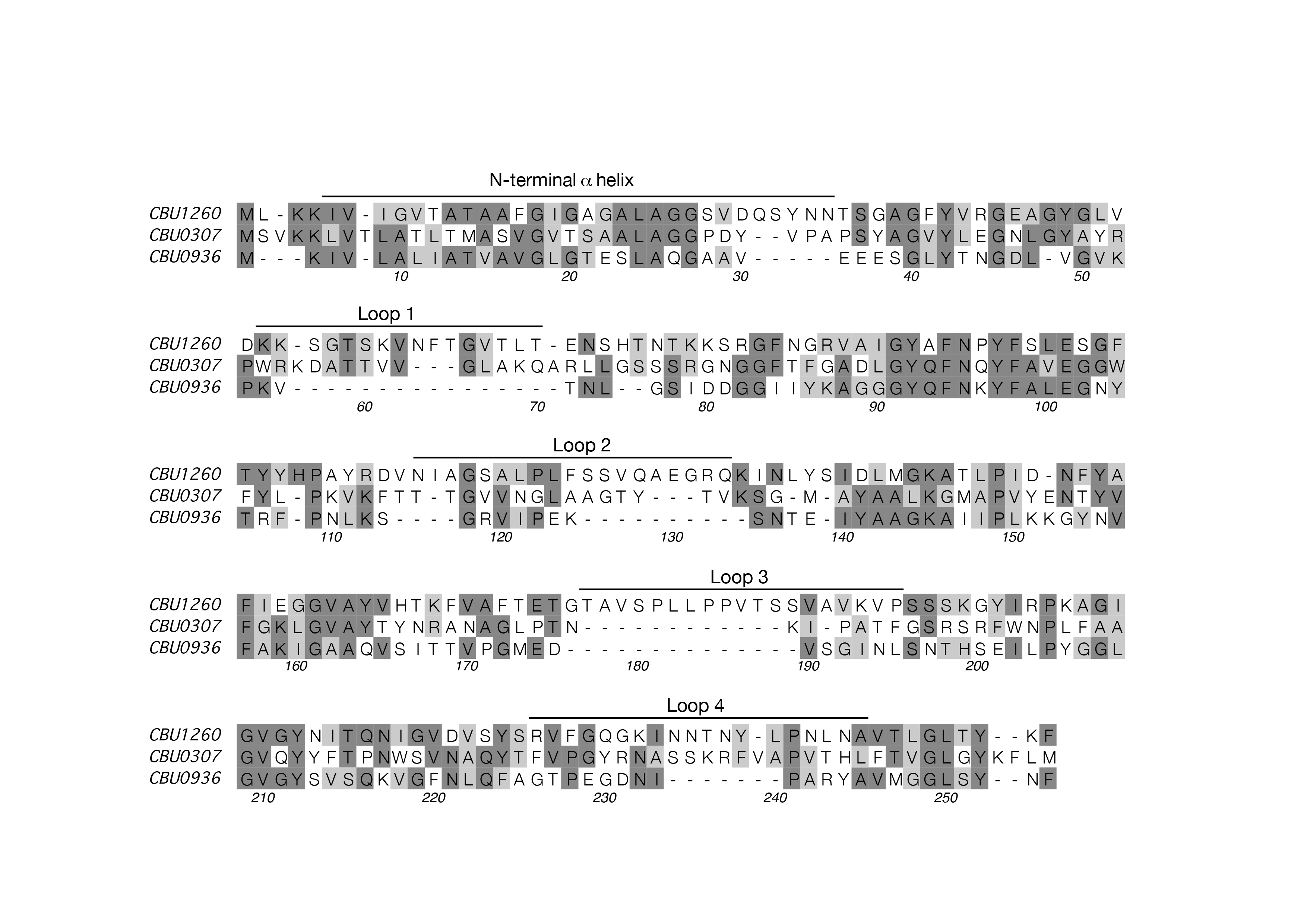

Supplement: Figure S7 — Sequence alignment of Coxiella OmpA-like transmembrane domain-containing proteins. The primary sequence of CBU_1260 (OmpA) was aligned to those of CBU_0307 and CBU_0936, two hypothetical proteins annotated as OmpA-like transmembrane domain-containing proteins. Light grey boxes indicate similarities, dark grey boxes indicate identity. (TIF) [file ppat.1004013.s007.tif]

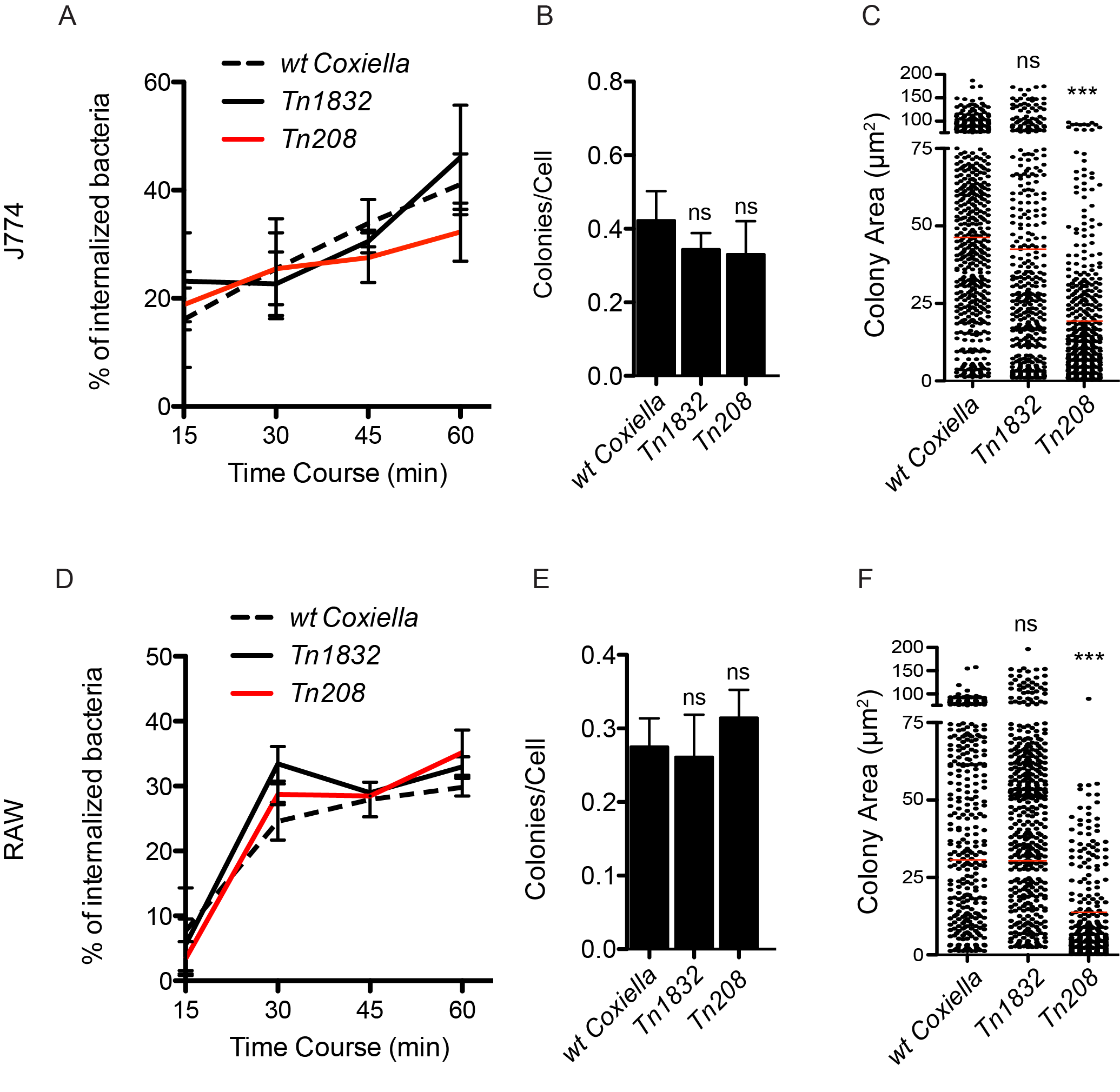

Supplement: Figure S8 — Coxiella internalization by J774 and RAW macrophages and intracellular replication. J774 (top charts) and RAW (bottom charts) macrophages were incubated with wt Coxiella, the control transposon mutant Tn1832 or the OmpA mutant Tn208 for the indicated time points. Cells were fixed and labeled with an anti-Coxiella antibody coupled to Alexa Fluor 555 and with Atto-647N phalloidin prior to cell permeabilization. Internalized bacteria were detected by GFP fluorescence in the case of Tn208 and Tn1832 whereas for wt Coxiella infections, cells were permeabilized and bacteria were stained with the anti-Coxiella antibody as above, coupled to Alexa Fluor 488. Alternatively, cells were fixed at 5 days after infection; DNA was labeled with Hoechst 33258 and wt Coxiella with the specific antibody as above. The automated image analysis software CellProfiler was used to calculate the percentage of internalized bacteria (A and D), the number of colonies/cell (B and E) and the area (in microns2) of intracellular Coxiella colonies (C and F) identified for each condition. Values are means ± standard deviations of triplicate experiments where an average of 8000 bacteria (A and D) or 400 vacuoles (B, C, E, F) were analyzed for each condition (values were compared to wt Coxiella infections. ns = non-significant; *** = P<0.001 2way ANOVA for A and D and t test for B, C, E, F). (TIF) [file ppat.1004013.s008.tif]

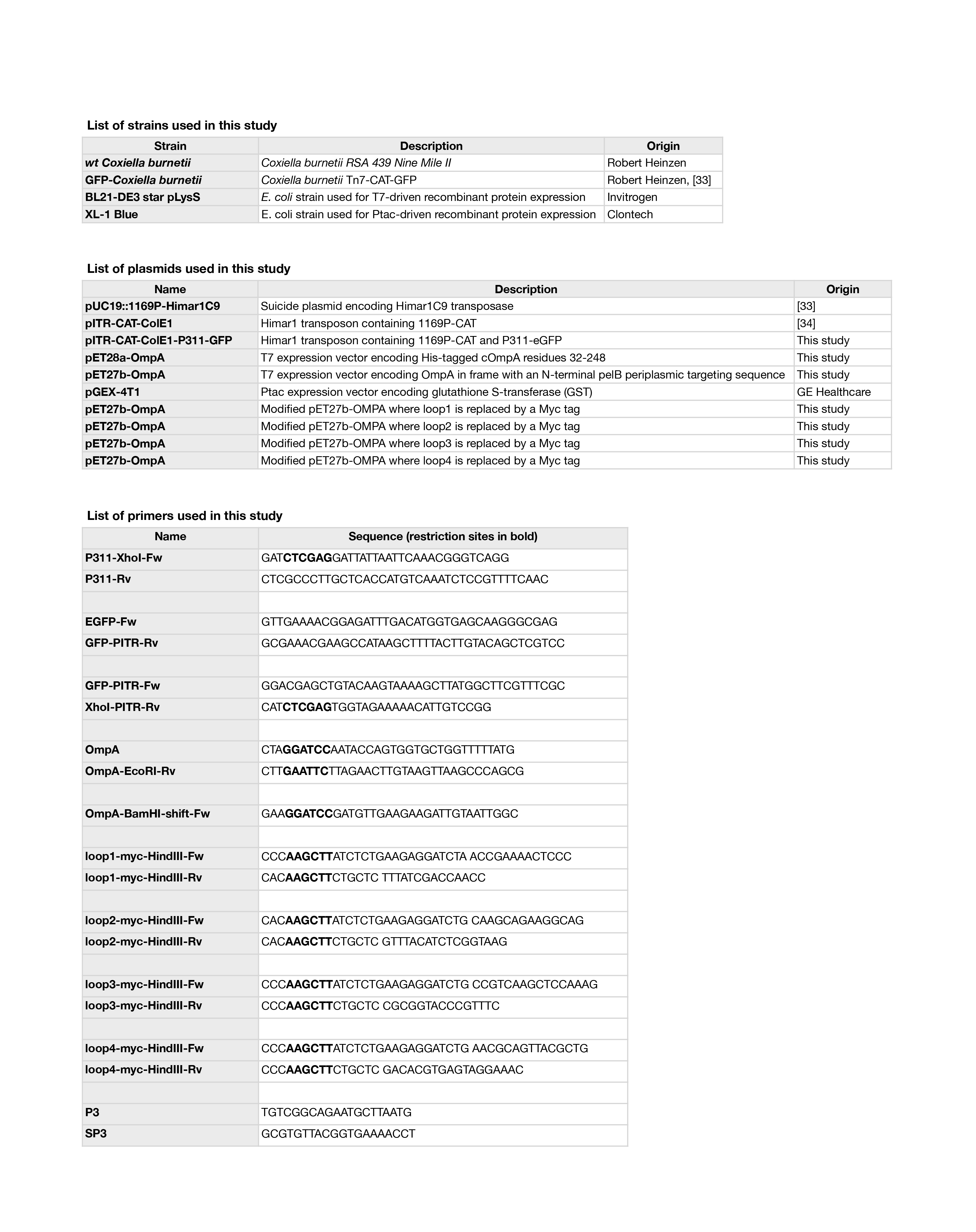

Supplement: Figure S9 — Tables of strains, plasmids and primers used in this study. (TIF) [file ppat.1004013.s009.tif]
